# Linalool enables reversible immobilization for high-resolution *in vivo* imaging during regeneration in *Schmidtea mediterranea*

**DOI:** 10.1016/j.isci.2026.117282

**Published:** 2026-09-04

**Authors:** Martijn Heleven, Charlotte Segnana, Bob Kauffmann, Reja Trippaers, Karolien Bijnens, Karen Smeets

**Affiliations:** 1 Zoology: Biodiversity and Toxicology, Centre for Environmental Sciences at Hasselt University, Agoralaan Gebouw D, 3590 Diepenbeek, Belgium

**Keywords:** linalool, live imaging, planarians, regeneration, immobilization

## Abstract

Live imaging in whole-organism models is often limited in both resolution and repeatability by the lack of relatively non-invasive, reversible immobilization methods. To enable real-time *in vivo* imaging in the highly regenerative planarian *S. mediterranea*, we systematically evaluated linalool as a sedation strategy. We identified 2.16 mM linalool as an effective concentration that induces rapid, reversible immobilization without compromising survival or causing detectable stress phenotypes. Acute exposure before amputation or repeated daily exposures during regeneration did not impair blastema growth, stem cell proliferation, or gross brain morphology, demonstrating compatibility with regeneration studies under imaging-relevant conditions. In contrast, continuous exposure throughout regeneration induced stress phenotypes and impaired regenerative outcomes. Together, our findings define operational exposure windows for linalool, establishing a robust, regeneration-compatible immobilization strategy that enables stable, high-resolution imaging across biological scales, from whole-organism organization to cellular dynamics, while remaining compatible with fluorescent dyes and allowing full recovery after imaging.

## Highlights

• Linalool enables stable, reversible immobilization for live imaging in planarians
• Imaging-compatible exposure paradigms preserve survival, regeneration, and tissue integrity
• Linalool enables high-resolution, multiplexed confocal imaging across biological scales

## Graphical abstract

**Figure:**
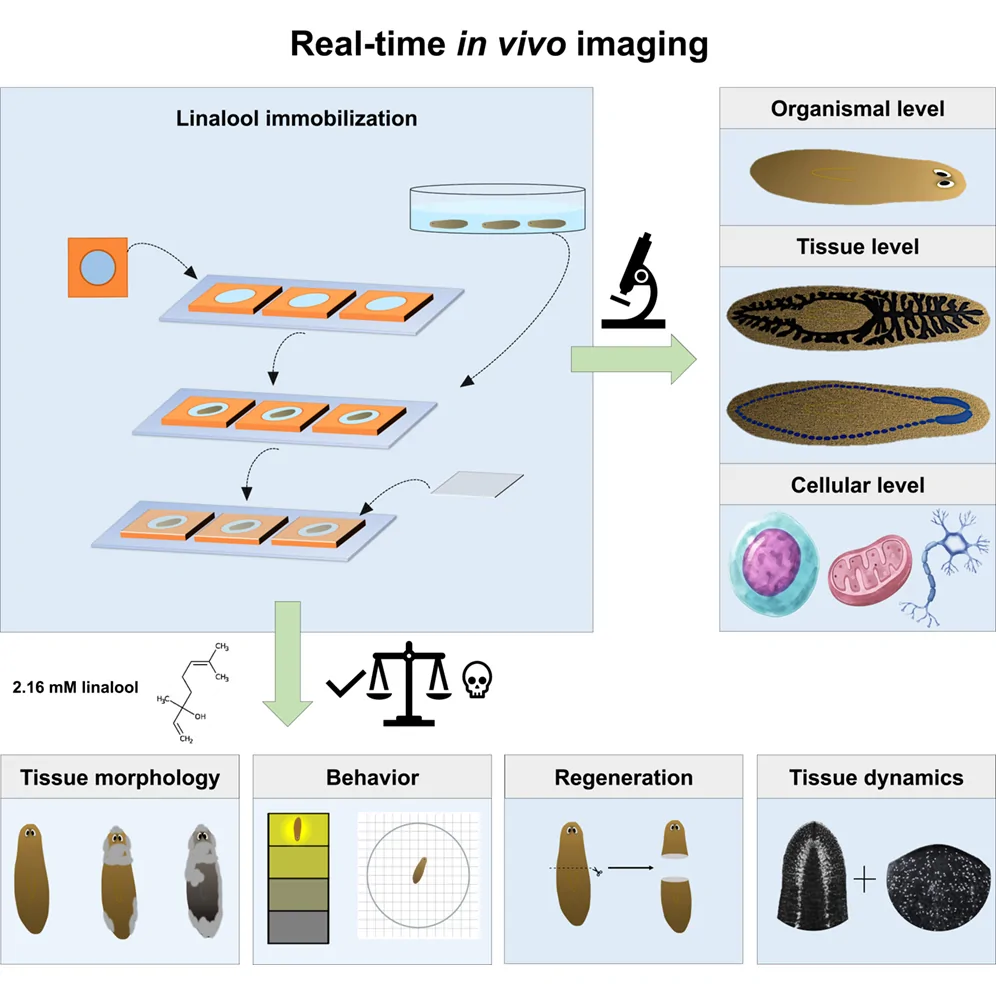

## Teaser

Developmental Biology; Environmental Toxicology; Live Imaging

## Introduction

Real-time *in vivo* imaging is indispensable for uncovering rapid or transient physiological responses that cannot be captured in isolated or fixed tissue preparations.^1^^,^^2^^,^^3^ Many biological processes, including development, tissue homeostasis, and regeneration, rely on dynamic cellular and molecular events that unfold on minute-to-hour timescales within tissues.^4^^,^^5^ Observing these processes in their native spatial context allows causal relationships to be established between early signaling responses and long-term functional outcomes.

Successful regeneration begins with immediate wound-induced signaling events occurring within minutes, followed by sustained cellular reprogramming, stem cell activation, tissue patterning, and organ reconstruction over days.^6^^,^^7^^,^^8^^,^^9^^,^^10^ This combination of rapid early responses and long-term structural remodeling necessitates repeated, high-resolution *in vivo* imaging of the same individual across multiple time points. Consequently, regeneration places specific demands on immobilization strategies, requiring them to be reversible, non-toxic, and physiologically well-tolerated.

Among the earliest responses is oxidative stress, which occurs within minutes after injury and is characterized by bursts of reactive oxygen species (ROS) and mitochondrial rearrangements in morphology and activity.^11^^,^^12^^,^^13^^,^^14^ Capturing these early events in real time is essential for linking molecular signaling dynamics to downstream regenerative outcomes.^9^^,^^12^

In vertebrate models such as zebrafish, real-time imaging has significantly advanced our understanding of regeneration by enabling the visualization of fast-acting and dynamic signaling events upon injury.^3^^,^^15^^,^^16^^,^^17^ Although these models have been invaluable for studying tissue plasticity, their regenerative capacity remains limited compared to certain invertebrate species.^4^^,^^18^ One such example is the freshwater planarian *Schmidtea mediterranea*, which can regenerate its entire body from small tissue fragments, including a fully functional central nervous system.^19^^,^^20^ Despite experimental advantages, such as their optical transparency, relatively simple anatomy, and expanding molecular toolkit, live imaging approaches in planarians remain underdeveloped.^21^^,^^22^^,^^23^^,^^24^ Advancing real-time imaging methodologies is, therefore, essential to fully exploit planarians as a powerful system to study large-scale tissue and organ regeneration at high spatial and temporal resolution.

A major limitation for real-time imaging in planarians is the lack of reliable, reversible, and non-toxic immobilization strategies. Current pharmacological approaches, such as tricaine, ethanol, or chloretone, are either non-reversible or induce physiological stress, neurotoxicity, or tissue damage.^25^^,^^26^^,^^27^^,^^28^^,^^29^ Together, these limitations highlight the need for a reversible immobilization strategy that enables stable imaging without interfering with physiology or regeneration. Although low-melting-point agarose is a useful, non-toxic immobilization method to perform wide-field fluorescent imaging of various dyes (e.g., ROS-sensitive dyes), its application is limited in more advanced imaging approaches. Residual internal tissue movements often persist within the agarose cavity, complicating high-resolution confocal imaging.^30^^,^^31^ Moreover, in high-throughput settings, agarose embedding is labor-intensive and may introduce optical scattering that compromises image quality in advanced microscopy pipelines.^31^^,^^32^ In addition, the requirement to handle animals in warm agarose can introduce technical constraints and operator-dependent variability, and if not carefully controlled, may affect tissue physiology. These drawbacks make long-term or repeated imaging of the same animal across regenerative time points challenging.

Linalool, a monoterpenoid alcohol commonly found in essential oils, has emerged as a potential alternative for reversible anesthesia in invertebrate models. In *Hydra*, low concentrations enable rapid immobilization with full recovery.^33^ In planarians, limited linalool usage has been reported, often in combination with low-melting-point agarose.^22^^,^^34^^,^^35^ While this suggests its potential as an immobilizing anesthetic, its independent application has not been systematically assessed. Before linalool can be broadly adopted for prolonged imaging applications, a systematic characterization of its efficacy-to-toxicity profile is required to assess its potential adverse physiological impact, determine optimal working concentrations, and evaluate its compatibility with reproducible imaging workflows. Here, we systematically evaluate the suitability of linalool as a reversible immobilization strategy for high-resolution *in vivo* imaging and characterize imaging-compatible exposure setups that are well tolerated across the regenerative endpoints assessed in this study.

## Results

### Concentration- and exposure-dependent toxicity of linalool

**Figure 1:**
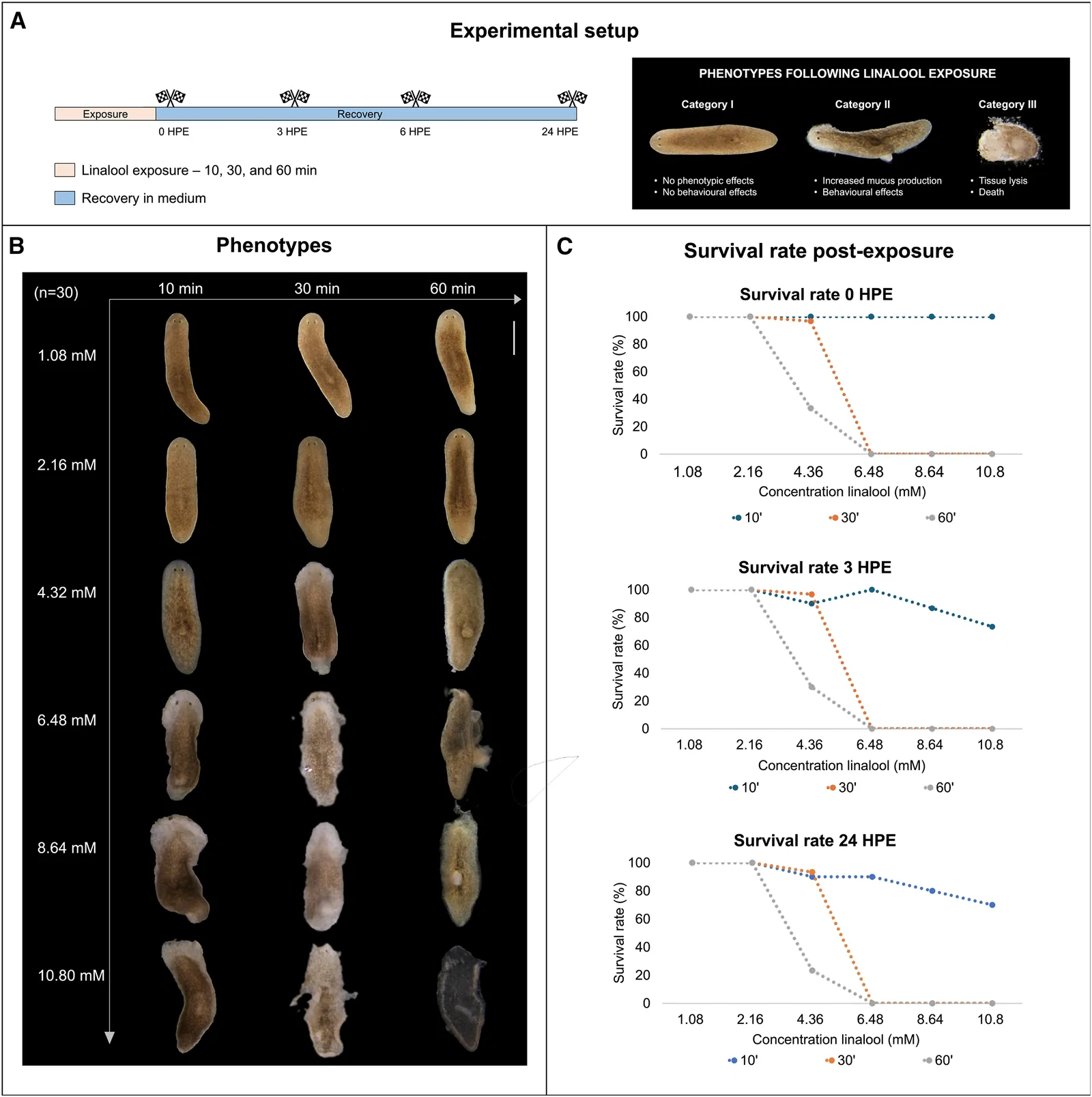
Concentration- and exposure-dependent toxicity of linalool (A) Graphical representation of the experimental setup. Animals were exposed for 10, 30, or 60 min and followed up for 24 h post exposure (HPE). At 3 HPE, 6 HPE, and 24 HPE, phenotype scoring of the animal was performed. Three different phenotype categories could be observed across conditions: (I) no observable phenotypical and behavioral effects; (II) visible increased mucus production and behavioral effects; (III) excessive tissue lysis and death. Successful immobilization was achieved when phenotype (I) co-occurred with little to no micromovements. (B) Representative phenotypes across conditions and exposure durations. Animals were exposed for 10, 30, or 60 min to concentrations ranging from 1.08 mM to 10.80 mM (*n* = 30 per condition). (C) Survival rate post exposure. Animals were followed up for 24 HPE. Survival rate (%) was quantified at 0, 3, and 24 HPE. Data represent mean % from three independent experiments. Scale bars, 2000 μm.

To determine the tolerable concentration range of linalool for live imaging, we assessed survival and phenotype following acute exposure to increasing concentrations for different exposure durations (Figure 1). Concentrations were selected as a stepwise incremental series to systematically define the transition between non-toxic and toxic conditions, including both commonly used application concentrations and higher doses.^22^^,^^34^^,^^35^ Phenotypes were classified into three categories (I–III) (Figure 1A).

At low concentrations (1.08–2.16 mM), no detectable gross morphological alterations could be observed across all exposure times (Figure 1B). At 4.32 mM, category II phenotypes, including increased mucus production and impaired motility, became apparent after 30–60 min of exposure, whereas concentrations ≥6.48 mM caused severe tissue damage and mortality (category III) in a duration-dependent manner.

Survival measurements confirmed a strong concentration- and time-dependent toxicity of linalool (Figure 1C). Immediately after exposure (0 h post exposure; 0 HPE), no mortality was observed at concentrations ≤2.16 mM under any exposure duration. At 4.32 mM, survival remained high after 10–30 min of exposure but declined severely after 60 min. At concentrations ≥6.48 mM, survival decreased sharply, with complete mortality observed after 30–60 min of exposure. In contrast, animals exposed for only 10 min showed 100% survival at all concentrations at 0 HPE.

Phenotypic follow-up during recovery showed that delayed mortality remained limited at low concentrations but increased at higher concentrations and longer exposure durations. At both 3 and 24 HPE, animals exposed to ≤2.16 mM survived across all exposure durations. In contrast, exposure to 4.32 mM or higher led to reduced survival during recovery (3–24 HPE), particularly after 30–60 min of exposure. Notably, after 24 HPE, a concentration-dependent decrease in survival was observed following 10 min of exposure, whereas longer exposures resulted in little or no survival from 6.48 mM onward.

### Linalool impairs motility while allowing complete recovery

To define imaging-compatible exposure windows, we assessed motility during and after exposure to non-lethal linalool concentrations.

Motility was evaluated under both standard and light-gradient conditions (Figures S1A and S1B), and decreased in a concentration-dependent manner. Control animals crossed on average ∼18 squares within 90 s, whereas animals exposed to 1.08 mM crossed fewer than half that number, and those exposed to 2.16 mM were effectively immobilized. During the first 2 min of exposure, animals frequently displayed transient body contractions, curling behavior, circular movements, or partial immobilization before reaching a more stable immobilized state. After approximately 2 min of exposure, animals exposed to 2.16 mM consistently exhibited stable immobilization, whereas responses at 1.08 mM were more variable, with most individuals retaining some locomotory capacity, albeit at a reduced level. The transition from the initial behavioral responses to effective immobilization is illustrated in a representative time-lapse in Video S1. Because an effective immobilization agent for imaging purposes should restrict animal displacement under defined stimuli, we assessed phototactic-based movement in planarians exposed to linalool. At 2.16 mM, motility under the light-gradient condition was significantly impaired. While controls spent ∼60 s in the darkest quadrant, linalool-treated animals largely failed to leave the initial high-light-intensity quadrant (Figure S1B).

Video S1. Behavioral responses of *Schmidtea mediterranea* during the initial minutes of exposure to 2.16 mM linalool, related to Figures S1 and S2

**Figure 2:**
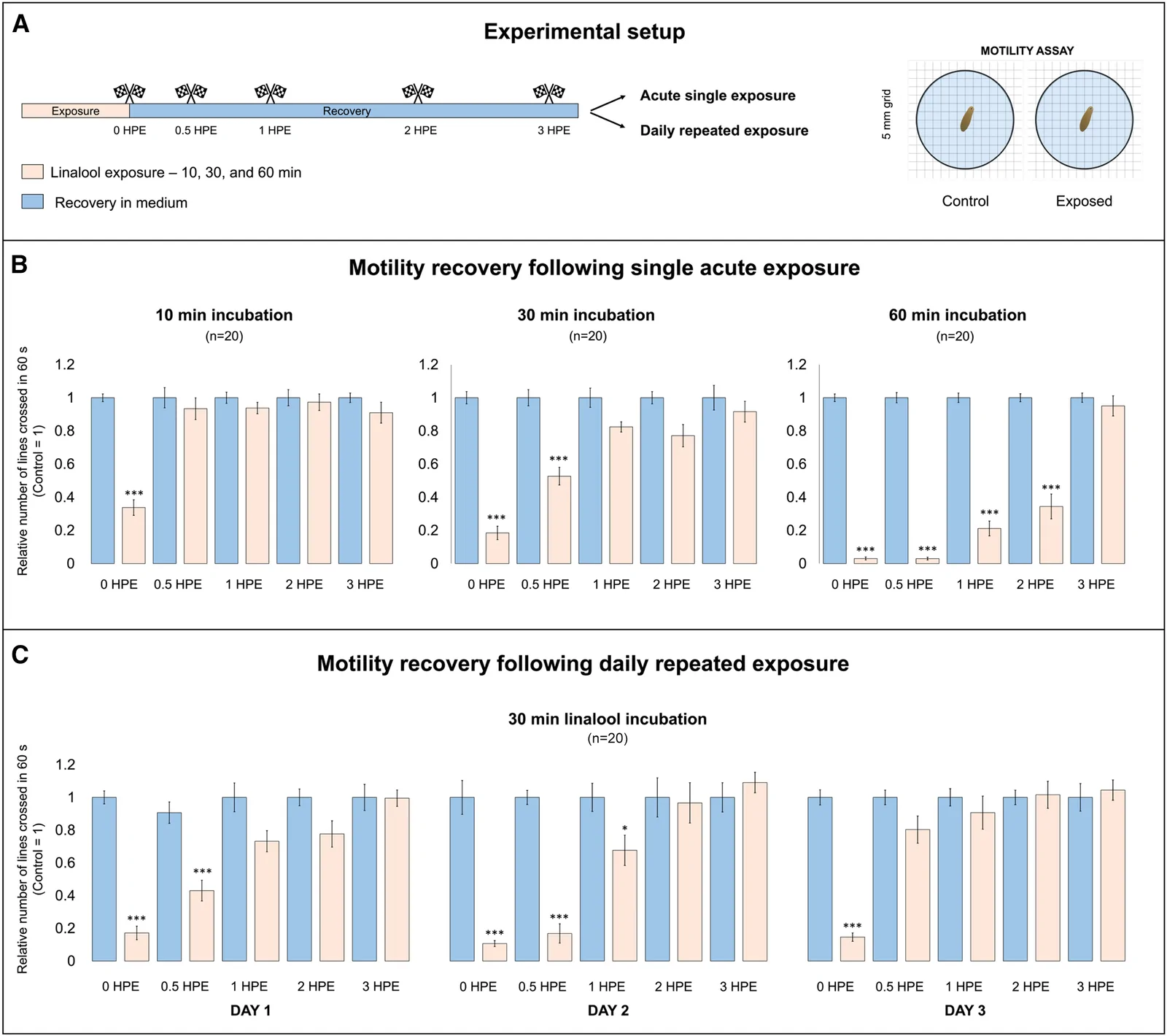
Motility recovery following acute and repeated linalool exposure (A) Graphical representation of the experimental setup. Planarians were exposed to 2.16 mM linalool for 10, 30, or 60 min (acute exposure) or to 30 min of linalool daily for up to 3 days (repeated exposure). Following transfer to fresh medium, motility recovery was assessed at 0, 0.5, 1, 2, and 3 h post-exposure (HPE) by quantifying the number of 5-mm grid lines crossed within 60 s. Values were normalized to untreated controls measured at the corresponding time point. (B) Motility recovery following single acute linalool exposures of 10, 30, or 60 min (*n* = 20 animals per condition). (C) Motility recovery following daily repeated 30 min of linalool exposure (*n* = 20 animals per condition). Data represent mean ± SEM from two independent experiments. Statistical significance was assessed using Wilcoxon rank-sum tests with Holm correction for multiple comparisons indicated by ∗*p* < 0.05, ∗∗*p* < 0.01, ∗∗∗*p* < 0.001.

To determine whether linalool-induced motility impairment was reversible under the imaging-relevant exposure conditions presented here, motility recovery was quantified following single acute exposures of 10, 30, or 60 min, as well as after repeated daily 30-min of exposures (Figure 2). Motility was assessed based on the number of grid lines crossed within 60 s and normalized to untreated controls for each time point. Following a single acute exposure, recovery kinetics depended on the duration of linalool incubation (Figure 2B). A 10-min exposure caused a clear reduction in motility immediately after treatment (0 HPE), after which animals rapidly recovered to control levels by 0.5 HPE and maintained normal motility thereafter. Increasing the exposure duration resulted in a slower recovery. Following a 30-min exposure, motility was strongly reduced at 0 HPE, partially recovered by 0.5 HPE, and further improved at 1–2 HPE, before returning to control levels by 3 HPE. The 60-min exposure induced the strongest immobilization, with virtually no movement immediately after treatment and at 0.5 HPE, followed by a slow recovery at 1–2 HPE and complete restoration of motility by 3 HPE.

To assess whether repeated exposure affected recovery, animals were exposed daily to 30 min of linalool for three consecutive days (Figure 2C). On each day, animals displayed a pronounced reduction in motility immediately after exposure, followed by progressive recovery during the subsequent hours. Motility consistently approached or reached control levels by 2–3 HPE. Importantly, repeated daily exposure did not delay recovery compared with a single 30-min exposure, indicating that no cumulative effects on locomotor function occurred.

### Short-term and continuous linalool exposure differentially affect regeneration

**Figure 3:**
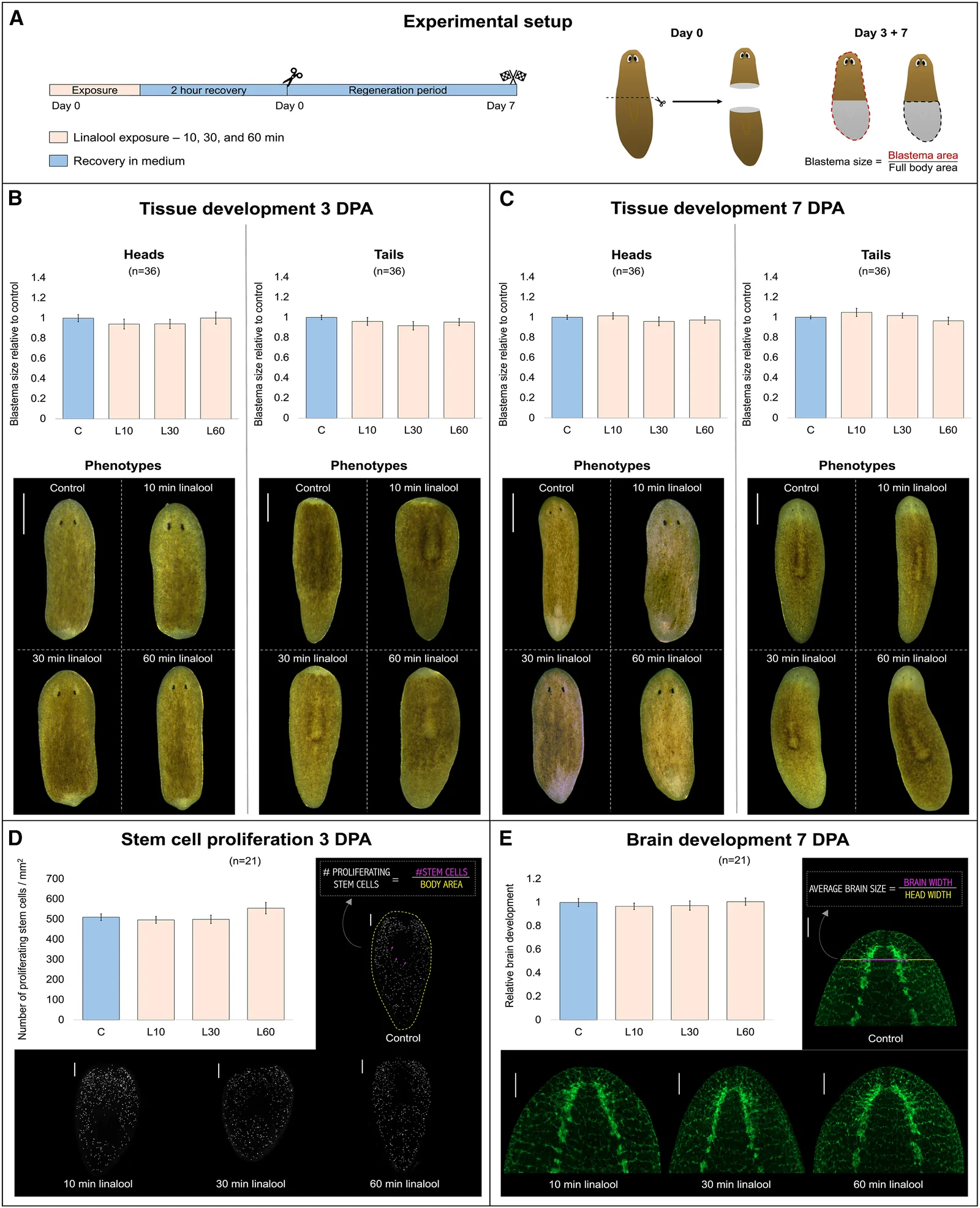
Acute linalool exposure does not affect regeneration (A) Graphical representation of the experimental setup. Animals were exposed to either medium or 2.16 mM linalool for 10, 30, or 60 min. After 2 h of recovery in medium, animals were transversaly amputated prior to the pharynx and allowed to regenerate in fresh medium. At 3 and 7 DPA, tissue development was scored by measuring the relative blastema size, which corresponds to the ratio of the total blastema area to the full body area. (B) Tissue development at 3 DPA (*n* = 36 per condition). Graphs represent the blastema size of regenerating heads and tails relative to the control following 10, 30, or 60 min of exposure. Representative phenotypes of each exposure duration are presented under each corresponding graph. Scale bars, 1000 μm. (C) Tissue development at 7 DPA (*n* = 36 per condition). Graphs represent the blastema size of regenerating heads and tails relative to the control following 10, 30, or 60 min of exposure. Representative phenotypes of each exposure duration are presented under each corresponding graph. Scale bars, 1000 μm. (D) Stem cell proliferation at 3 DPA in regenerating tails (*n* = 21 per condition). The graph represents the number of proliferating stem cells normalized to the total body area. Representative images of regenerating tails are shown for each exposure duration. Scale bars, 100 μm. (E) Brain development at 7 DPA in regenerating tails (*n* = 21 per condition). The graph represents the relative gross brain size following 10, 30, or 60 min of exposure. Average brain size was calculated by the ratio of the total brain width to the total head width. Representative images of regenerating tails are shown for each exposure duration. Scale bars, 100 μm. Data represent mean ± SEM from three independent experiments. Statistical significance compared to the control was assessed using unpaired Student’s *t* tests with Holm correction for multiple comparisons. ∗*p* < 0.05, ∗∗*p* < 0.01, ∗∗∗*p* < 0.001.

**Figure 4:**
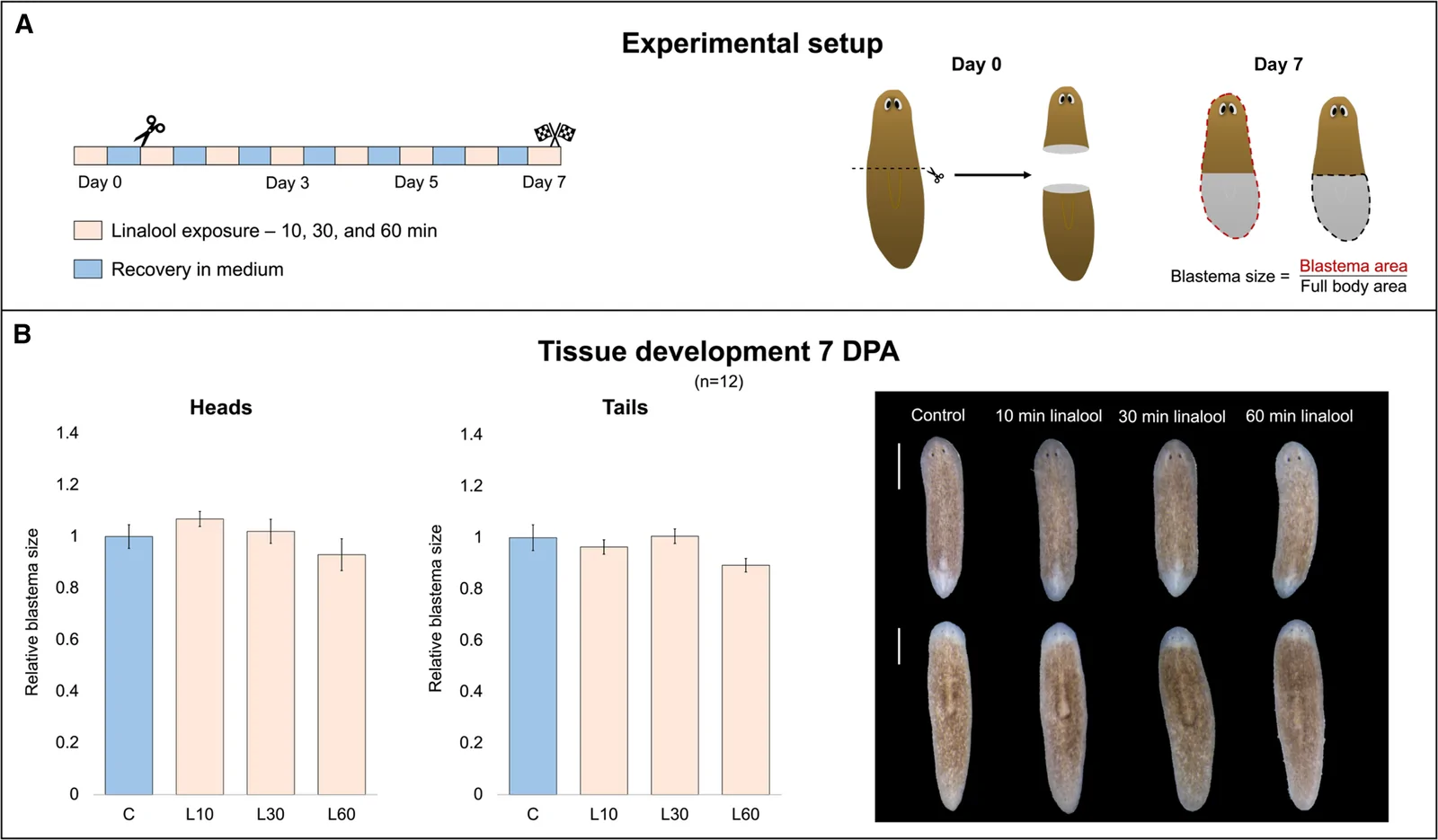
Daily repeated linalool exposure does not affect regeneration (A) Graphical representation of the experimental setup. Animals were exposed for 10, 30, or 60 min to 2.16 mM linalool, after which they were placed in medium to recover for 2 h. Next, animals were transversaly cut prior to the pharynx to create a regenerating head and tail. Regenerating tissue fragments were exposed daily and washed afterward for up to 7 days. At 7 DPA, tissue development was scored by measuring the relative blastema size, which corresponds to the ratio of the total blastema area to the full body area. (B) Tissue development at 7 DPA (*n* = 12 per condition). Graphs represent the relative blastema size of regenerating heads and tails compared to the control. Representative images of regenerating heads and tails are shown for each exposure duration. Scale bars, 1000 μm. Data represent mean ± SEM from two independent experiments. Statistical significance compared to the control was assessed using Welch’s *t* tests with Holm correction for multiple comparisons. ∗*p* < 0.05, ∗∗*p* < 0.01, ∗∗∗*p* < 0.001.

To determine whether 2.16 mM linalool affects regenerating animals, we assessed regeneration capacity following short-term exposure to linalool. Animals were exposed once prior to amputation or repeatedly during regeneration for 10, 30, or 60 min per day (Figure 3; Figure 4). Acute pre-amputation exposure altered neither blastema size nor proliferation at 3 days post amputation (DPA), nor gross brain morphology at 7 DPA (Figures 3B–3E). Similarly, repeated daily exposures across all imaging-relevant durations up to 7 DPA did not impair blastema formation in head or tail fragments (Figure 4B).

To test whether exposure duration affects regenerative outcomes, we next investigated the effects of continuous linalool exposure throughout regeneration up to 5 DPA (Figure S2). In contrast to the imaging-relevant short-term exposures, continuous treatment resulted in pronounced regenerative defects, causing a significant concentration-dependent reduction in tissue growth at 5 DPA in both head and tail fragments. Regenerating tail fragments additionally displayed category II phenotypes, including tissue damage (Figure S2B).

### Linalool enables stable real-time *in vivo* imaging across biological scales

**Figure 5:**
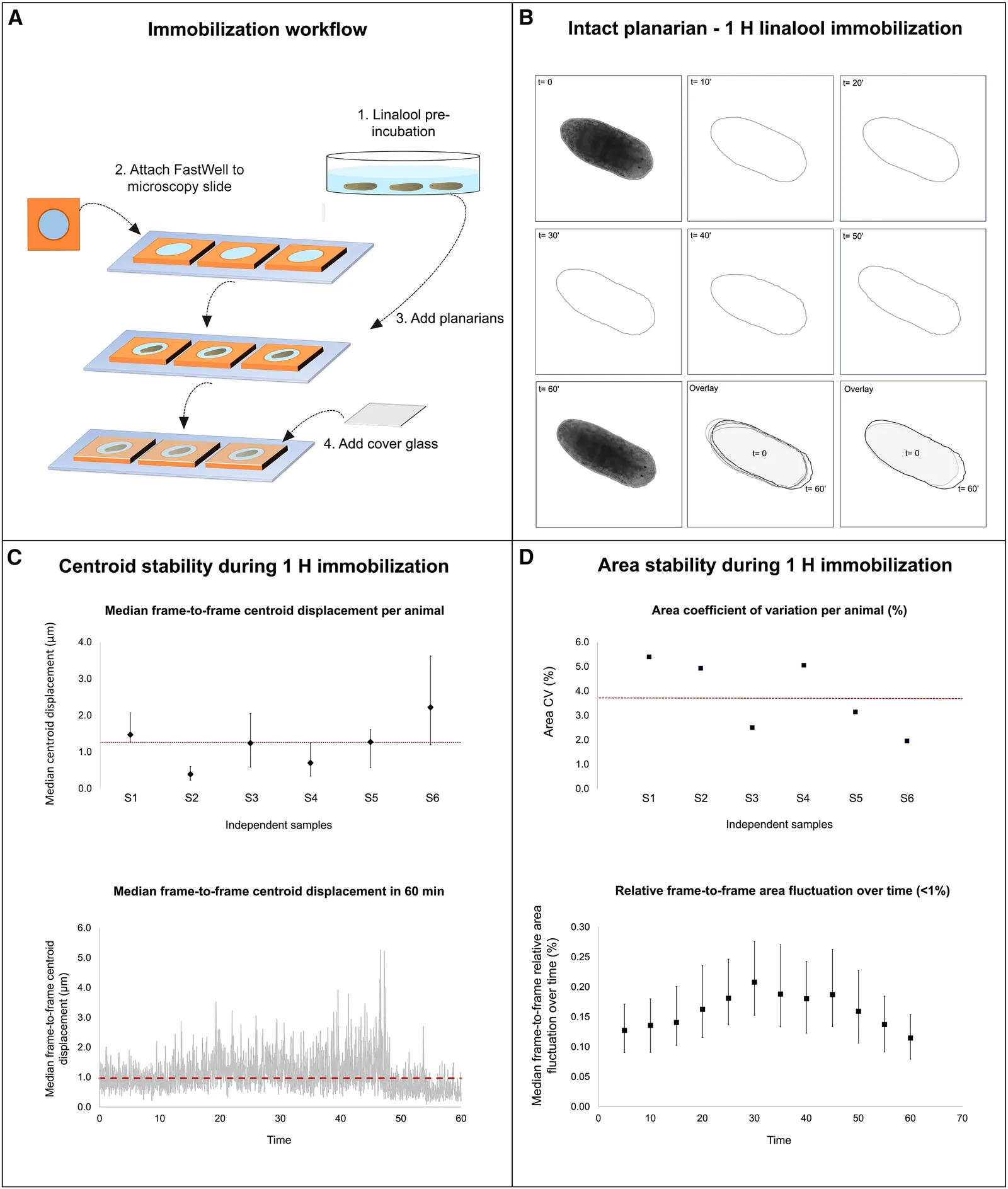
Linalool allows stable immobilization for real-time *in vivo* imaging (A) Graphical representation of the immobilization workflow. Animals were pre-incubated with 2.16 mM linalool for up to 2 min in a closed Petri dish, after which they were transferred in a droplet of linalool to a FastWell attached to a microscopy slide. Next, a cover glass was added with slight pressure to create a sealed imaging chamber. Depending on the region of interest (ROI) or the fluorescent label, the microscopy slide can be replaced by a long coverslip to allow rotation to scan both dorsal and ventral ROIs. (B) Intact planarians during 1 h immobilization with 2.16 mM linalool. Immobilized samples were followed up for 1 h by taking 1 frame/second brightfield snapshots (*n* = 6). A representative sample is shown over time by highlighting its outline every 10 min. Total overlay is presented for all seven time points next to the start (t = 0) and endpoint (t = 60 min) overlay. (C) Centroid stability during 1 h linalool immobilization. The upper graph shows the median frame-to-frame centroid displacement per animal (μm) of six independent samples. The lower graph shows the median frame-to-frame centroid displacement over 60 min. (D) Area stability during 1 h linalool immobilization. The upper graph shows the area coefficient of variation (%) per animal. The lower graph shows the relative frame-to-frame area fluctuation over time. Data represent median ± interquartile range (IQR) from six independent samples.

To validate linalool as an effective immobilization strategy for continuous live imaging, animals were pre-incubated in 2.16 mM linalool for up to 2 min and mounted in linalool in FastWells (Figure 5A). This workflow enabled stable continuous immobilization for at least 1 h, allowing real-time brightfield imaging at 1 frame per s of intact and regenerating animals across multiple biological scales.

At the whole-organism level, brightfield imaging of intact animals, acquired at 1 frame s^−1^ for 60 min, revealed only minimal displacement despite their higher baseline motility compared with regenerating fragments (Figures 5B and 5C). Analysis of centroid position relative to the initial frame (t = 0) showed that 98.23 % of all frames deviated by less than 30 μm over the 1 h imaging period (Figures S3A and S3B). During the first 10 min, all frames remained within 10 μm of the starting position, increasing to approximately 15 μm by 30 min and reaching up to 30 μm during the final 30 min, indicating a slow cumulative drift of the animal’s centroid over time (Figure S3C).

In contrast, frame-to-frame analysis revealed only minimal short-timescale displacement (Figure 5C). Median frame-to-frame centroid displacement was ≤1.26 μm in four out of six animals, resulting in an overall median frame-to-frame displacement of 0.96 μm across samples (Figure 5C). These values indicate that residual movements remained minimal, supporting stable high-temporal-resolution imaging.

Consistent with these observations, area-based measurements further confirmed positional stability (Figure 5D). The median area coefficient of variation per sample was below 4 % in three out of six animals, while the median frame-to-frame relative area fluctuation over time remained below 0.2 %, indicating minimal residual shape or area changes during imaging (Figure 5D). Importantly, these measurements were obtained under a high-frequency imaging regime (1 frame per s), representing a stringent acquisition setting, and, therefore, suggest that residual variation would be even lower during imaging at longer time intervals.

**Figure 6:**
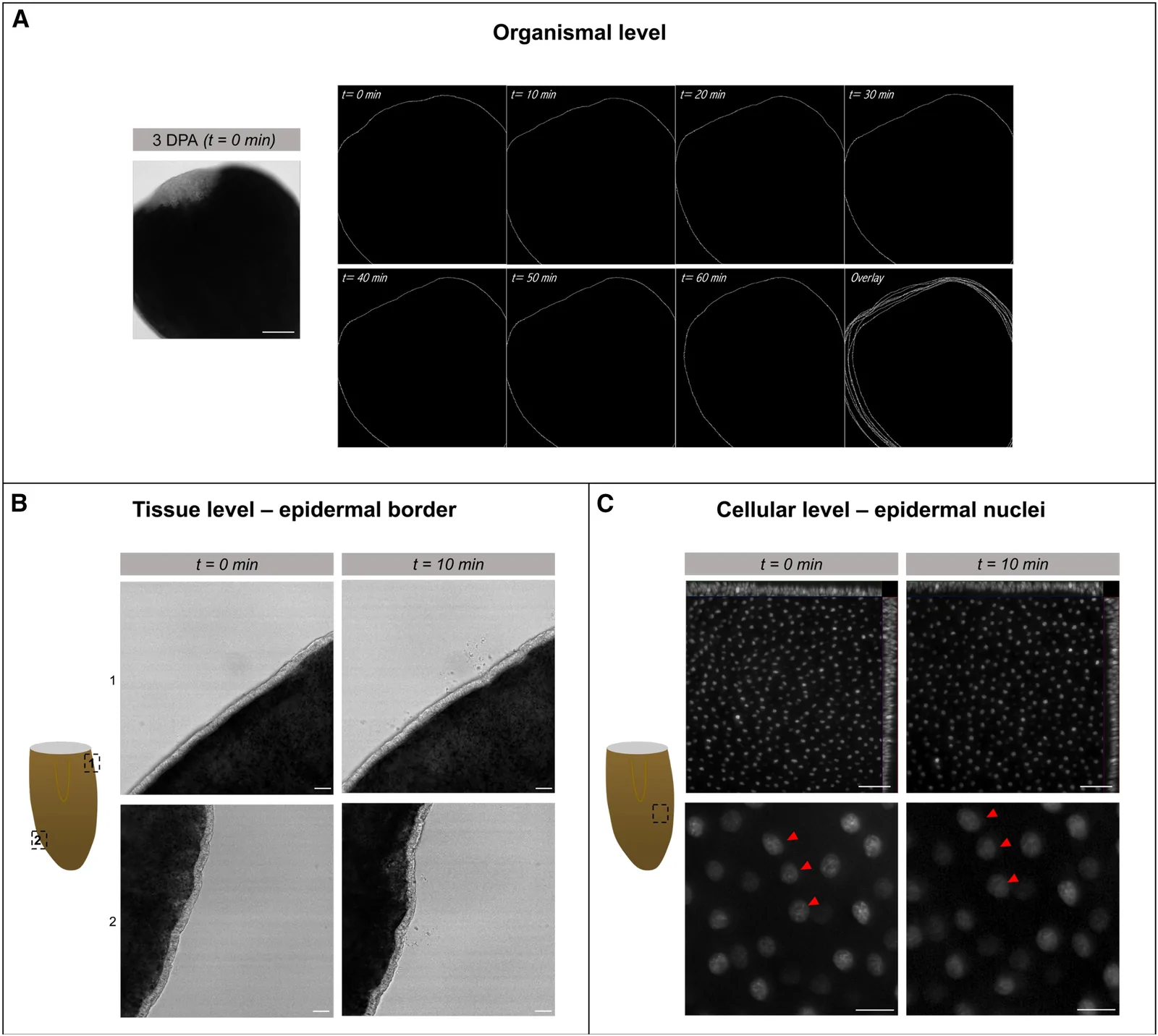
Real-time *in vivo* imaging during regeneration using linalool (A) Linalool allows imaging at the organismal level. Regenerating tail at 3 DPA imaged with brightfield microscopy (10× objective) for 1 h using linalool. The outline of the animal is shown every 10 min, and an overlay overtime is presented in the last frame (t = time in min). Scale bars, 200 μm. (B) Linalool allows imaging at the tissue level. Epidermal border of a regenerating tail imaged for 10 min with brightfield microscopy (20× objective). Scale bars, 50 μm. (C) Representative confocal images of nuclei in a regenerating tail fragment stained with Hoechst and acquired at t = 0 and t = 10 min using a 40× water-immersion objective. Images are presented as maximum-intensity projections of 24 optical sections spanning a total z-depth of 23 μm (approximately two cell layers). Scale bars, 10 μm.

At the tissue and cellular levels, linalool treatment allowed continuous tracking of the epidermal border and epidermal nuclei for at least 10 min (Figures 6B and 6C). Z-stacks acquired before and after continuous 405 nm excitation at 1 frame per s for 10 min confirmed that individual nuclei could be consistently visualized at high magnification in living animals, demonstrating that linalool immobilization supports stable real-time imaging at cellular resolution *in vivo* (Figure 6C).

### Linalool-based *in vivo* imaging is compatible with diverse fluorescent probes

**Figure 7:**
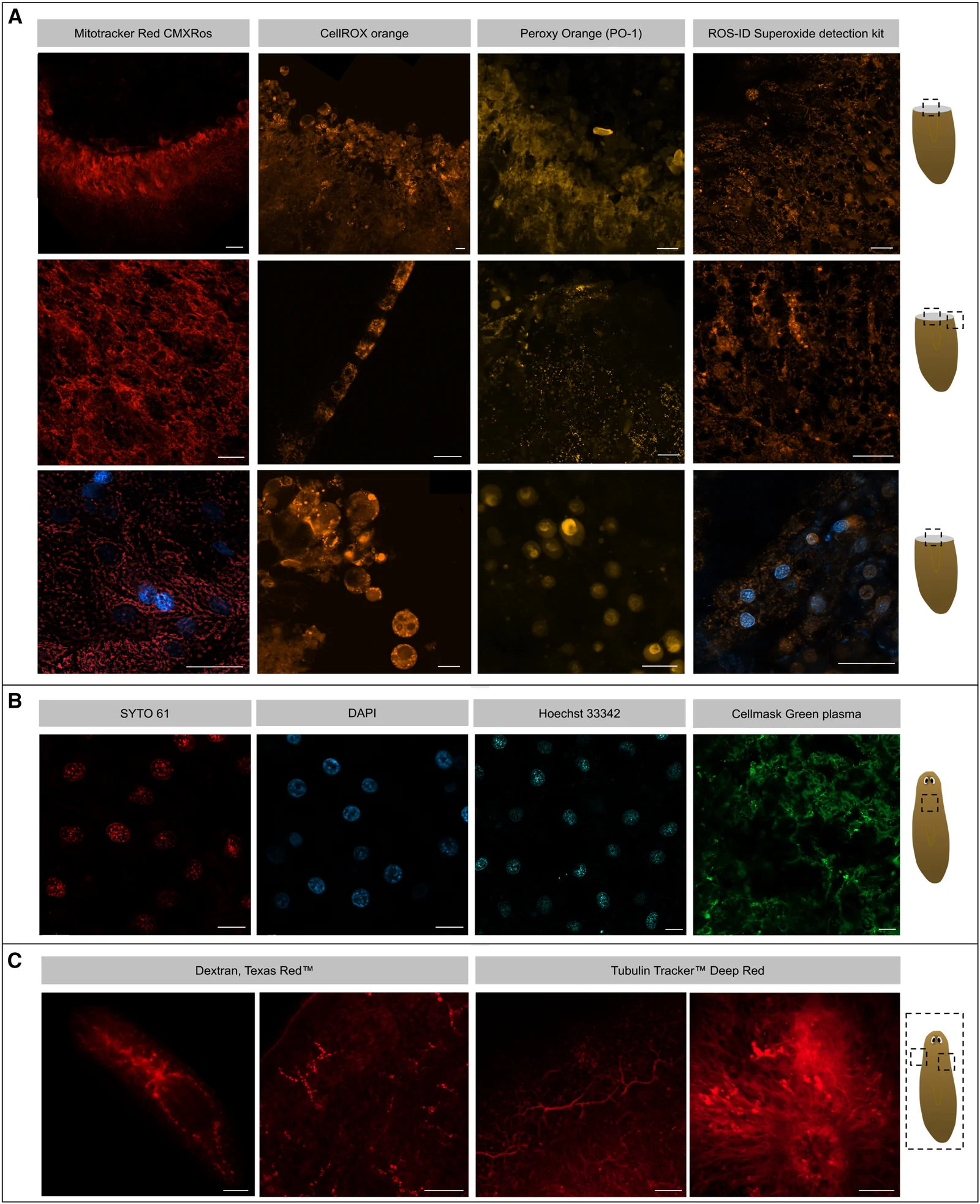
Linalool immobilization allows high-resolution confocal microscopy (A) Representative confocal images demonstrating compatibility with mitochondrial and redox-associated probes. From left to right: MitoTracker Red CMXRos revealing mitochondrial networks, CellROX Orange detecting general reactive oxygen species (ROS), Peroxy Orange (PO-1) visualizing hydrogen peroxide, and the ROS-ID Superoxide Detection Kit detecting superoxide. Images illustrate visualization across multiple biological scales from top to bottom. Tissue-level wound responses were acquired using a 20× objective, except for the superoxide images, which were acquired using a 40× water-immersion objective. Scale bars, 50 μm. Tissue-specific localization images were acquired using a 20× objective, except for the CellROX Orange and superoxide images, which were acquired using a 40× water-immersion objective. Scale bars, 20 μm. Cellular-resolution images were acquired using a 40× water-immersion objective, except for the PO-1 images, which were acquired using a 20× objective. Scale bars, 20 μm. (B) Representative images of cellular labeling. Nuclei were visualized using SYTO 61, DAPI, and Hoechst 33342, while CellMask Green labeled plasma membranes across multiple cell types. All images were acquired using a 40× water-immersion objective with Airyscan processing. Scale bars, 10 μm (SYTO 61, DAPI, and Hoechst 33342) and 20 μm (CellMask Green). (C) Representative examples of organism- and tissue-level labeling. Injection of Dextran Texas Red into the intestinal lumen or head mesenchymal tissue enabled visualization of the intestinal tract and excretory structures using a 10× objective. Tubulin Tracker Deep Red labeled tubulin-positive structures within the subepidermal body wall (40× water-immersion objective) and at the injection wound (10× objective). Scale bars, 100 μm (Dextran overview) and 20 μm (Dextran detail and Tubulin Tracker images).

**Figure 8:**
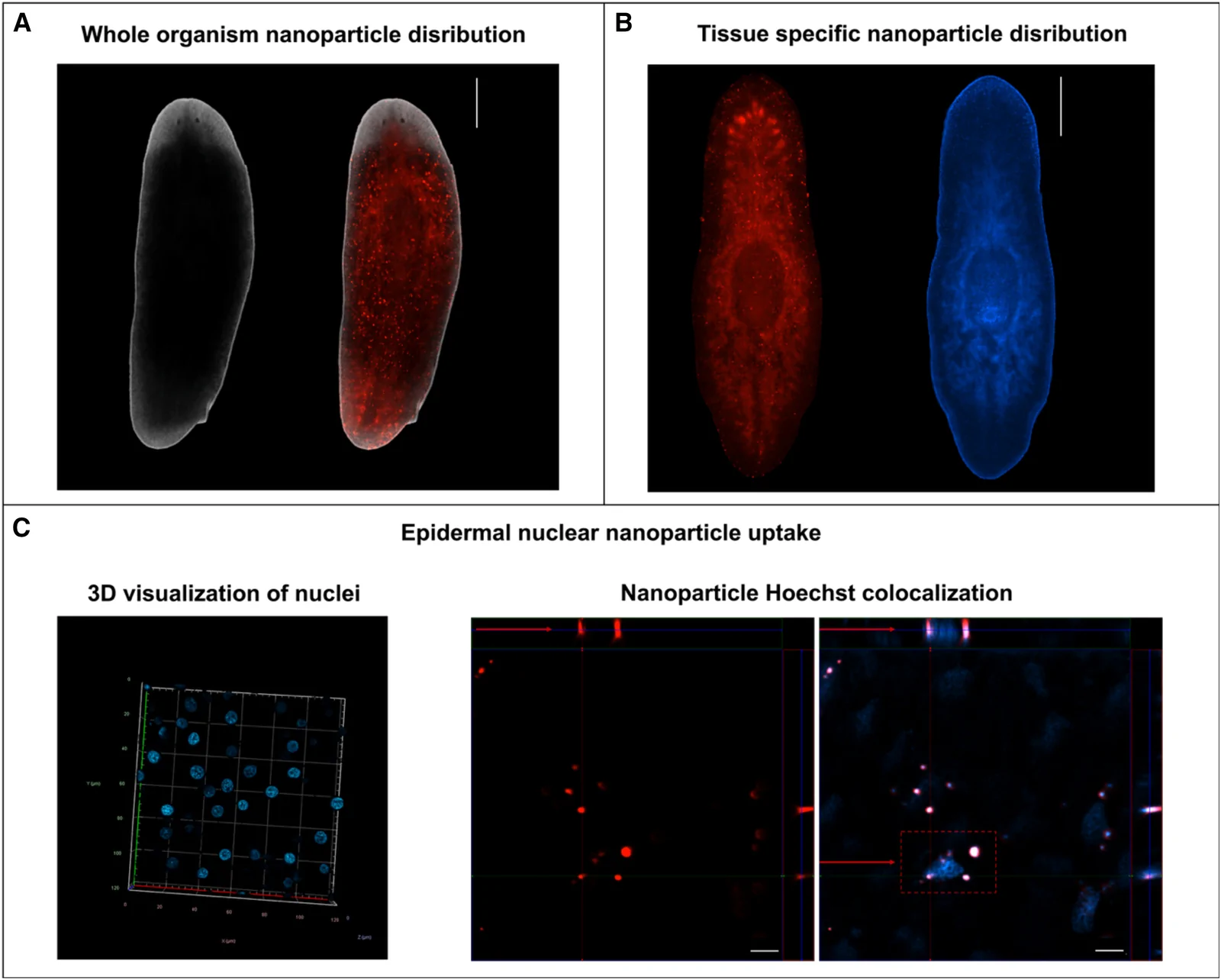
Linalool immobilization allows uptake visualization of nanoplastics (A) Nanoplastic uptake at the organismal level. Brightfield image of an intact planarian acquired using a 10× objective (left) and the corresponding overlay with red fluorescent 200 nm polystyrene nanoparticles (right). Scale bars, 500 μm. (B) Overview images acquired using a 10× objective showing the distribution of red fluorescent 200 nm polystyrene nanoparticles, with prominent accumulation in the intestinal tract (left), and Hoechst nuclear staining used for colocalization experiments (right). Scale bars, 500 μm. (C) Left: representative confocal z-stack acquired using a 40× water-immersion objective to enable in-depth colocalization analysis at the cellular level. Nuclei were stained with Hoechst and imaged as 59 optical sections spanning a total z-depth of 12.76 μm. Right: representative Airyscan image showing colocalization of red fluorescent 200 nm polystyrene nanoparticles (red) with epidermal nuclei stained with Hoechst (blue). The image is presented as an orthogonal projection of a z-stack consisting of 23 optical sections spanning a total z-depth of 5.28 μm, acquired using a 40× water-immersion objective. Scale bars, 5 μm.

To evaluate whether linalool immobilization supports high-resolution live imaging with commonly used probes, we tested a range of fluorescent dyes targeting dynamic signaling molecules, cellular structures, and tissues, as well as fluorescent nanoplastics. Stable real-time imaging was achieved across all probe classes (Figure 7 and 8; Table S1), demonstrating broad compatibility of linalool-based immobilization with high-resolution live fluorescence imaging.

To evaluate compatibility with dynamic signaling molecules, we first examined several redox-associated probes (Figure 7A). MitoTracker Red CMXRos enabled visualization of mitochondrial networks, whereas CellROX Orange detected general ROS. In addition, Peroxy Orange (PO-1) and the ROS-ID Superoxide Detection Kit specifically visualized hydrogen peroxide and superoxide, respectively. Interestingly, these probes enabled imaging across multiple biological scales. At the tissue level, all probes produced strong fluorescence signals at the wound site 1 h post amputation. At higher magnification, tissue- and location-specific signals became apparent, revealing ROS-associated fluorescence in multiple cell types, including epidermal cells located at the body margin near the wound. Cellular-resolution imaging further resolved individual ROS-positive cells surrounding the wound, while PO-1 additionally revealed prominent nuclear H_2_O_2_-associated fluorescence.

At the cellular level, nuclei could be visualized using SYTO 61, DAPI, and Hoechst 33342, while CellMask Green labeled plasma membranes across cell types (Figure 7B). Combined with the redox-associated probes shown previously, these findings demonstrate that linalool immobilization supports high-resolution multiplexed fluorescence imaging, enabling simultaneous visualization of multiple cellular structures and signaling molecules within the same living animal.

At the tissue level, injection of Dextran Texas Red into the intestinal lumen enabled visualization of the gut, whereas mesenchymal injections additionally labeled excretory structures in the head region (Figure 7C). Tubulin Tracker Deep Red successfully labeled tubulin-positive structures within the subepidermal body wall and at the injection site, further illustrating the compatibility of linalool immobilization with high-resolution imaging across different tissue compartments and experimental setups.

Finally, linalool immobilization also proved compatible with imaging of fluorescent nanoplastics. As a proof of concept, fluorescent 200 nm polystyrene nanoparticles were visualized *in vivo*, revealing widespread uptake throughout the animal (Figure 8). High-resolution confocal z-stack acquisition combined with Hoechst nuclear staining enabled multiplexed visualization of intracellular fluorescent nanoplastic uptake in epidermal cells, while orthogonal projections confirmed nuclear uptake (Figure 8C).

## Discussion

Real-time imaging in living organisms has become an indispensable tool for uncovering dynamic physiological processes within their natural multicellular context. In regeneration research, identifying early wound-induced signals is critical for causally linking signaling events and transcriptional changes to specific regenerative outcomes.^4^^,^^8^^,^^19^ However, capturing the earliest responses following amputation requires high-resolution imaging at the cellular level, complementing the whole-body signaling insights already established in regenerating planarians at fixed time points. Examples of these signals include transient bursts of ROS and calcium that occur at the wound site, making real-time *in vivo* imaging indispensable for understanding their dynamic roles in regeneration.^12^^,^^13^^,^^36^^,^^37^

Planarians, such as *Schmidtea mediterranea*, are a powerful model for investigating fundamental aspects of tissue regeneration and developmental biology, but their exceptional regenerative capacity remains challenging to study due to limited live-imaging approaches. The absence of stable transgenic lines constrains genetic labeling approaches that are routine in other systems, such as *Danio rerio* or *Drosophila melanogaster*.^22^^,^^23^^,^^38^^,^^39^ Nonetheless, a wide range of fluorescent dyes, tracers, and small-molecule probes has been successfully employed in other model organisms to visualize processes, such as oxidative stress, calcium signaling, mitochondrial activity, and cytoskeletal dynamics.^11^^,^^40^^,^^41^^,^^42^^,^^43^ Extending these applications to planarians, and visualizing them in real time at both the tissue and cellular levels rather than only at the whole-body scale, requires immobilization methods that are reversible, non-toxic, and compatible with high-resolution imaging workflows.

Here, we establish linalool as a practical and versatile immobilization strategy that enables high-resolution live imaging in planarians. Previous reversible immobilization approaches relied on low-melting-point agarose embedding, a workflow that is labor intensive, limits experimental throughput, and has primarily been applied to widefield imaging or visualization of epidermal nuclei at relatively low spatial resolution.^31^^,^^32^^,^^34^^,^^44^ Likewise, although recently developed microfluidic devices enable prolonged observation of regenerating planarians, they provide limited immobilization and currently do not permit high-resolution cellular imaging.^45^ To address these limitations, we systematically evaluated linalool as a standalone immobilization agent. We show that 2.16 mM linalool induced rapid and reversible immobilization, effectively suppressing motility while maintaining high survival and complete recovery within 3 h following acute and repeated daily exposure (Figures 1, 2, and S1). These immobilizing properties are observed across diverse organisms, although their potency and optimal application might be species dependent. Goel and colleagues demonstrated that 1 mM linalool produces rapid and reversible immobilization in *Hydra* without affecting regeneration and cell proliferation, closely mirroring the reversible effects we observed after repeated exposure for 10, 30, or 60 min (Figure 4). In contrast, studies in zebrafish and carp have reported only modest sedative effects of linalool, highlighting species-specific differences in potency and application.^46^^,^^47^

Besides its effective and reversible immobilizing properties, linalool enables visualization across biological scales, from whole-organism organization to cellular resolution, while substantially reducing motion artifacts that typically complicate live imaging in planarians (Figure 6). Quantitative analysis revealed only a gradual cumulative drift during prolonged imaging (Figures 5 and S3), whereas frame-to-frame displacements remained minimal, with median values below 1.3 μm across samples (Figures 5C and 5D). Given that the average planarian nuclear diameter is approximately 5 μm, these residual movements are well within a range that can be readily corrected computationally, supporting reliable high-resolution imaging at the cellular scale. These findings establish and validate a regeneration-compatible linalool exposure paradigm for both short-term and longitudinal live imaging. By combining daily imaging sessions of up to 1 h with complete recovery between sessions over at least 3 consecutive days, this approach spans the critical early phases of regeneration, including both major stem cell proliferation peaks (Figures 2–4).^19^ Consequently, it provides a robust experimental framework for following early signaling events after amputation to subsequently link them to cellular behaviors and regenerative outcomes within the same regenerating individual.

A key application of the validated imaging paradigm is the reliable use of the extensive repertoire of fluorescent probes routinely employed in other model organisms, whose application in live planarians has remained limited.^6^^,^^12^^,^^30^^,^^44^^,^^48^ Here, we demonstrate that linalool-based immobilization is broadly compatible with probes targeting dynamic signaling molecules, cellular structures, tissue architecture, and fluorescent nanomaterials (Figures 7 and 8). Moreover, the successful combination of multiple fluorescent probes, including nuclear markers with redox probes, demonstrates that linalool-based immobilization supports multiplexed imaging approaches and high-resolution confocal z-stack acquisition. Together, these findings substantially expand the experimental toolkit available for studying dynamic physiological processes and intracellular localization events in living planarians.

Beyond its technical advantages and species-dependent effects, it is important to consider the physiological effects and chemical stability of linalool. Despite being a plant-derived monoterpene with reported antioxidant properties and neuro-modulating capacities, neither acute nor repeated short-term exposure interfered with the regenerative parameters assessed, including gross brain morphology (Figure 3).^49^^,^^50^^,^^51^^,^^52^ Nevertheless, more subtle effects on specific physiological readouts cannot be excluded. We did observe severe regenerative defects, including tissue damage and lysis, following continuous linalool exposure throughout regeneration (Figure S2), indicating that its use should be restricted to acute or repeated short-term treatments. Additionally, careful control of environmental parameters, including the type and airtightness of the immobilization chamber, is essential to ensure reproducibility, as linalool can undergo oxidation to products with reduced sedative properties.^53^^,^^54^ We further recommend adapting chamber depth to planarian size to position the ventral surface as close as possible to the coverslip and working with water-immersion objectives to optimize the working distance, thereby maximizing image quality during live imaging.

Together, our findings establish linalool as a versatile strategy for high-resolution and multiplexed live imaging in *S. mediterranea*. By enabling stable imaging across biological scales and compatibility with a broad repertoire of fluorescent probes, linalool substantially expands the experimental toolkit available for regeneration and toxicology research. As genetically encoded reporters, biosensors, and advanced optical imaging modalities such as light-sheet microscopy become increasingly available for planarians, this straightforward immobilization strategy provides a practical foundation for studying regeneration in living organisms with increased spatial and temporal resolution.

### Limitations of the study

Although linalool exhibited sedative properties in *Schmidtea mediterranea* under the exposure paradigms investigated here, its applicability to other species requires species-specific optimization of concentration and exposure duration, which can be done based on the workflow and quantitative validation framework presented in this study. In addition, factors such as animal size and prolonged exposure conditions were not systematically evaluated and may influence immobilization efficacy or recovery dynamics. Finally, the present study was not designed to directly benchmark linalool against existing immobilization approaches, and dedicated comparative studies evaluating immobilization efficiency, fluorophore compatibility, and imaging performance across methodologies will be valuable to further define the strengths and limitations of linalool-based immobilization strategies. Nevertheless, our study provides a quantitative framework to assess linalool in regenerative imaging studies and demonstrates its compatibility with several imaging-relevant regenerative endpoints, supporting its use as an accessible and versatile tool for high-resolution *in vivo* imaging in the freshwater planarian *Schmidtea mediterranea*.

## Resource availability

### Lead contact

Requests for further information and resources should be directed to and will be fulfilled by the lead contact, Martijn Heleven (martijn.heleven@uhasselt.be).

### Materials availability

The study did not generate new, unique reagents.

### Data and code availability

• Data reported in this paper will be shared by the lead contact upon request.
• No new code was generated in this study.
• Any additional information required to reanalyze the data reported in this paper is available from the lead contact upon request.

## Acknowledgments

The authors thank Natascha Steffanie and Ria Vanderspikken for their skillful technical assistance, and Silke Wilms for her contribution during her internship. This work was supported by the 10.13039/501100007229Special Research Fund (BOF) of Hasselt University, the Research Foundation 10.13039/501100011878Flanders (10.13039/501100003130FWO, project nos. G047921N and 11N0825N), and the European Union’s 10.13039/100018693Horizon Europe Research and Innovation Programme through the Marie Skłodowska-Curie Actions (MSCA-DN, grant agreement no. 101073238). The research presented in this publication was conducted using infrastructure funded by the European Marine Biological Resource Centre (EMBRC) Belgium through the FWO project no. I000825N.

## Author contributions

M.H., conceptualization, methodology, validation, formal analysis, investigation, writing – original draft preparation, writing – review and editing, visualization; C.S., investigation, writing – review and editing; B.K., investigation, writing – review and editing; R.T., investigation, writing – review and editing; K.B., writing – review and editing; K.S., conceptualization, resources, writing – review and editing, supervision, project administration, funding acquisition.

## Declaration of interests

The authors declare no conflict of interest.

## STAR★Methods

### Key resources table

**Table:** 

REAGENT or RESOURCE	SOURCE	IDENTIFIER
**Antibodies**		

Mouse Anti-Synapsin Monoclonal Antibody (anti-SYNORF1), Unconjugated	Developmental Studies Hybridoma Bank	Cat# 3C11; RRID: AB_528479
Rabbit Anti-Phospho-Histone H3 (Ser10) (D2C8)	Cell Signaling	Cat# 3377S; RRID: AB_2799431
Goat anti-Mouse IgG (H + L) Cross-Adsorbed Secondary Antibody, Alexa Fluor™ 568 Conjugated	Molecular Probes	Cat# A-11004; RRID: AB_2534072
Goat anti-Rabbit IgG (H + L) Cross-Adsorbed Secondary Antibody, Alexa Fluor™ 488 Conjugated	Molecular Probes	Cat# A-11008; RRID: AB_143165

**Chemicals, peptides, and recombinant proteins**		

Peroxy Orange 1 (PO1)	Sigma-Aldrich	Cat#SML0688
CellROX™ Orange Reagent	Fisher Scientific	Cat#12807322
Invitrogen™ CellROX™ Deep Red Reagent	Invitrogen™	Cat#12013765
MitoTracker™ Red CMXRos	Invitrogen™	Cat#11569106
SYTO™ 61 Red Fluorescent Nucleic Acid Stain	Thermo Fisher Scientific	Cat#S11343
DAPI	Invitrogen™	Cat#11580246
Hoechst 33342, trihydrochloride trihydrate	Thermo Fisher Scientific	Cat#H3570
Tubulin Tracker™ Deep Red	Invitrogen™	Cat#T34077
CellMask™ Plasma Membrane Stains	Invitrogen™	Cat#C37608
Invitrogen™ Dextran, Texas Red™, 70,000 MW, Lysine Fixable	Invitrogen™	Cat#11540236
200 nm red fluorescent polystyrene NPs	Magsphere Inc	Cat#PSF-200NM

**Critical commercial assays**		

ROS-ID® Superoxide detection kit	Enzo Life Sciences	Cat#ENZ-51012

**Experimental models: Organisms/strains**		

*Schmidtea mediterranea*	This paper	N/A

**Software and algorithms**		

Fiji (ImageJ)	Fiji	RRID:SCR_002285
R Project for Statistical Computing	R Development Core Team	RRID:SCR_001905
RStudio	Team RStudio	RRID:SCR_000432
ZEISS ZEN Microscopy Software	ZEISS	RRID:SCR_013672

### Experimental model and study participant details

For all experiments, an asexual strain of the freshwater planarian *Schmidtea mediterranea* originally obtained from the University of Barcelona and maintained in the Smeets laboratory for over 10 years was used. Age was not controlled as an experimental variable, as it was not considered a relevant parameter under the experimental conditions used in this study. All experiments were initiated using fully regenerated animals to ensure a consistent physiological state. No ethical approval was required because *S*. *mediterranea* is an invertebrate species. Animals were continuously cultured at 20 °C in planarian medium consisting of ultrapure H_2_O supplemented with 1.6 mM NaCl, 1 mM CaCl_2_, 1 mM MgSO_4_, 0.1 mM MgCl_2_, 0.1 mM KCl, and 1.2 mM NaHCO_3_. Animals were fed veal liver once per week and continuously kept in the dark.

### Method details

#### General experimental setup

Unless otherwise stated, experiments were performed using animals that were starved for 7 days prior to experimentation. Intact planarians were amputated transversely between the photoreceptors and the pharynx to generate regenerating head and tail fragments (Figure S4). Following amputation, fragments were transferred to fresh planarian or exposure medium and allowed to regenerate under standard culture conditions at 20 °C. Regeneration time points are reported as hours post amputation (HPA) or days post amputation (DPA). Animals were randomly assigned to experimental treatments and maintained in 6-well plates containing fresh solutions. After linalool treatment, animals were rinsed three times with fresh medium and maintained under standard culture conditions for downstream analyses. Experiments included 6–10 animals per condition, depending on the assay, and were repeated in at least two independent biological replicates. Sample sizes varied between assays and are reported in the corresponding figure legends.

#### Linalool exposure

To identify a linalool concentration compatible with live imaging applications, we assessed both viability and immobilization efficiency. Linalool (≥97% purity; Acros Organics) was supplied in an amber glass bottle, stored at room temperature (RT) protected from light, and working concentrations were always freshly prepared by dilution in planarian medium prior to each experiment. Unless otherwise stated, planarians were exposed in groups at a density corresponding to 1 mL exposure medium per animal. Based on the density (0.861 g mL^−1^) and molecular weight of linalool (154.25 g mol^−1^), pure linalool corresponds to a theoretical concentration of approximately 5.41 M. Working concentrations were selected based on previous reports employing linalool as an anesthetic in planarians and other aquatic model systems.^22^^,^^33^^,^^34^

Planarians were first exposed to 1.08, 2.16, 4.32, 6.48, 8.64, or 10.8 mM linalool for 10, 30, or 60 min to evaluate concentration- and time-dependent effects on survival. Survival and tissue integrity were assessed immediately following exposure and again at 3, 6, and 24 h post exposure (HPE). Phenotypes were classified into three categories: category I (no observable effects on morphology and motility), category II (increase in mucus production, affected motility, and/or tissue lysis), and category III (excessive mucus production, severe tissue lysis and mortality). The optimal concentration (2.16 mM) was further tested for durations exceeding 60 min, which is relevant for extended live imaging. Planarians were incubated continuously in 2.16 mM linalool and monitored every 30 min for up to 24 h for signs of disintegration or loss of structural integrity.

To evaluate the suitability of 2.16 mM linalool for imaging applications relevant to regeneration studies, three exposure approaches and two amputation approaches were investigated. Exposure approaches consisted of acute exposure, repeated exposure, and continuous exposure. Acute and repeated exposure experiments were performed in parafilm-sealed well plates to minimize evaporation of the volatile compound, whereas continuous exposure experiments were intentionally not sealed to maintain oxygen availability during regeneration and were instead kept in a closed container with daily medium refreshment. Animals were either amputated following a 2 h recovery period after linalool exposure or amputated directly in linalool-containing medium. For acute exposure, intact planarians were submerged in 2.16 mM linalool for 10, 30, or 60 min. After treatment, animals were rinsed three times with fresh medium and allowed to recover for 2 h before being amputated transversely between the pharynx and the photoreceptors. This recovery period prior to amputation was included, as planarian motility was largely or fully restored by this time point across all exposure conditions and to reduce the likelihood of potential carry-over effects of acute linalool exposure. Animals were subsequently allowed to regenerate in fresh medium to assess potential effects on regeneration.

For the repeated exposure condition, animals underwent the same initial exposure, recovery, and amputation procedure but were subsequently exposed daily to 2.16 mM linalool for the respective durations until 7 DPA.

Finally, the effects of continuous exposure were investigated to assess the consequences of sustained linalool exposure during both amputation and regeneration. Animals were pre-exposed to 50, 100, or 500 μM linalool, amputated directly in linalool-containing medium, and continuously maintained in their respective exposure solutions until 5 DPA. Exposure solutions were refreshed every 24 h.

#### Motility analyses

The effectiveness of linalool as a reversible immobilizing agent was evaluated using motility assays. More specifically, planarian motility was assessed with a 1 min grid-crossing test adapted from Lowe and colleagues.^55^ Animals were placed in petri dishes overlaid with millimetre grid paper, filled with either control medium or 2.16 mM linalool. After a 2 min acclimation period, the number of 5 × 5 mm grid lines crossed in 60 s was counted. Motility under a light-gradient condition was assessed using the light-avoidance assay.^56^ For this, animals were placed in a rectangular container divided into four sections with a lamp (2650 K) positioned above the first section. After an adaptation period of 2 min post linalool exposure, animals were positioned in the container and the light avoidance ability was quantified as the time spent in the section furthest from the light during a 90-s interval.

#### Morphological characterization

To evaluate the effects of linalool exposure on tissue integrity (phenotype) or regenerative capacity, images were acquired with a Nikon DS-Ri2 digital camera attached to a Nikon SMZ800 stereomicroscope. The effect on the regenerative capacity was evaluated by comparing differences in blastema sizes at 3 and 7 days post amputation. Blastema and planarian sizes were determined using ImageJ (version 1.53g) and blastema size was normalized to the total body area of each planarian to account for minor differences in animal size.

#### Whole-Mount immunohistochemistry

Potential effects on stem cell dynamics and CNS development were assessed in whole-mount immunostained regenerating planarians at 3 DPA and 7 DPA, respectively. Animals were chilled on ice to induce body extension and treated with 2% HCl in Milli-Q water for 5 min to remove mucus and euthanize them. For synapsin and phospho-histone H3 staining, animals were fixed using 4% formaldehyde and Carnoy’s solution, respectively, after which they were processed as described previously.^6^ Primary antibodies were Mouse anti-synapsin (3C11, 1:50) and Rabbit anti-phospho-histone H3 (1:1000), followed by secondary antibodies Alexa Fluor goat anti-mouse 488 (1:400) and Alexa Fluor goat anti-rabbit 568 (1:500), respectively. Primary antibody incubation steps were performed for either 40 h (H3P primary) or 18–24 h (synapsin primary) at 4 °C, while secondary antibodies were always incubated for 3 h at RT. Samples were mounted in ImmuMount and imaged using a Zeiss LSM 900 confocal microscope. Ganglia width and mitotic cell number were quantified using ImageJ (version 1.53g) and normalized to total head width and body size, respectively.

#### Application of linalool for *in vivo* imaging

In the established and optimized linalool-based imaging setup, 2.16 mM linalool was used and prepared in fresh planarian medium. For real-time *in vivo* detection of dynamic processes (e.g., reactive oxygen species, mitochondrial network formation), planarians were transversely cut between the pharynx and the photoreceptors and incubated for 1 h in a range of fluorescent dyes at concentrations listed in Table S1. One day prior to imaging, fluorescent dextrans were injected into the prepharyngeal part of the gut of intact worms, using a Nanoject II (Drummond Scientific) to visualize the intestinal tract at the tissue level, while part of the excretory system was also stained when injected in the head mesenchymal tissue as described previously.^31^^,^^57^ Following staining, animals were quickly washed with fresh medium and exposed to 2.16 mM linalool in a Parafilm-sealed 6-well plate until no movement was observed (typically within 2 min). For imaging, immobilized planarians were placed with the ventral side up in a droplet of 2.16 mM linalool in a FastWell chamber, or similar setup depending on the size of the planarian, adhered to a microscope slide. A coverslip was then used to seal the chamber, enabling stable imaging from the ventral side. Animals were imaged using the Zeiss LSM 900 confocal microscope in both widefield and LSM mode (ZEISS). Where applicable, confocal z-stacks were acquired and visualized as orthogonal views. The number of optical sections and z-step intervals used for each experiment are indicated in the corresponding figure legends.

#### Microscopy

Samples were imaged using a Zeiss LSM 900 confocal microscope equipped with an Axio Observer 7 inverted stand, a 120 V HXP illuminator, and GaAsP-PMT or multialkali PMT detectors. The following objectives were used depending on the application: EC Plan-Neofluar 10×/0.30 NA, Plan-Apochromat 20×/0.80 NA, and LD C-Apochromat 40× Water/1.10 NA. Fluorophores were excited using 405 nm (5 mW), 488 nm (10 mW), 561 nm (10 mW), or 640 nm (5 mW) diode lasers. For confocal imaging, a pinhole diameter of 1 Airy unit (AU) was used throughout all experiments.

### Quantification and statistical analysis

Time-lapse recordings were acquired using a Zeiss LSM900 confocal microscope with transmitted light (bright-field) illumination. Images were recorded at a rate of 1 frame s^−1^ for 60 min to monitor positional and morphological stability during immobilization. Total animal movement stability was quantified by analyzing the centroid position of each animal across time. For each frame, the outline of the animal was automatically threshold-segmented to obtain a binary mask, from which centroid coordinates were extracted. Frame-to-frame centroid displacement was calculated from centroid coordinates of consecutive frames. The median centroid displacement per animal was used as a summary measure of positional stability.

To assess morphological stability during immobilization, the projected body area of each segmented animal was measured across the time-lapse sequence. Variability in body shape was quantified using the coefficient of variation (CV) of body area for each animal. In addition, relative frame-to-frame area fluctuations were calculated by measuring changes in body area between consecutive frames and normalizing these values to the mean body area of each animal. Image segmentation and measurements were performed using Fiji/ImageJ. Measurements were obtained from six independent animals imaged under identical conditions.

All statistical analyses were conducted in RStudio (version 2024.04.2 + 764). Data normality was assessed using the Shapiro–Wilk test. For comparisons between two independent groups, unpaired Student’s *t*-tests or Welch’s *t*-tests were used for normally distributed data, depending on the equality of variances. When assumptions of normality could not be met despite square-root or logarithmic transformation, the Wilcoxon rank-sum test was applied for pairwise comparisons. Holm correction was applied to account for multiple testing where appropriate. Statistical significance was defined as *p* < 0.05. The statistical test used for each experiment is indicated in the corresponding figure legends.

## References

[1] Zattara E.E., Turlington K.W., Bely A.E. (2016). Long-term time-lapse live imaging reveals extensive cell migration during annelid regeneration. BMC Dev. Biol..

[2] Jones J.D., Ramser H.E., Woessner A.E., Quinn K.P. (2018). In vivo multiphoton microscopy detects longitudinal metabolic changes associated with delayed skin wound healing. Commun. Biol..

[3] Greenspan L.J., Ameyaw K.K., Castranova D., Mertus C.A., Weinstein B.M. (2024). Live Imaging of Cutaneous Wound Healing after Rotary Tool Injury in Zebrafish. J. Invest. Dermatol..

[4] Poss K.D., Tanaka E.M. (2024). Hallmarks of regeneration. Cell Stem Cell.

[5] Niethammer P. (2016). The early wound signals. Curr. Opin. Genet. Dev..

[6] Pirotte N., Stevens A.S., Fraguas S., Plusquin M., Van Roten A., Van Belleghem F., Paesen R., Ameloot M., Cebrià F., Artois T., Smeets K. (2015). Reactive Oxygen Species in Planarian Regeneration: An Upstream Necessity for Correct Patterning and Brain Formation. Oxid. Med. Cell. Longev..

[7] Zhang Q., Wang Y., Man L., Zhu Z., Bai X., Wei S., Liu Y., Liu M., Wang X., Gu X., Wang Y. (2016). Reactive oxygen species generated from skeletal muscles are required for gecko tail regeneration. Sci. Rep..

[8] Owlarn S., Klenner F., Schmidt D., Rabert F., Tomasso A., Reuter H., Mulaw M.A., Moritz S., Gentile L., Weidinger G., Bartscherer K. (2017). Generic wound signals initiate regeneration in missing-tissue contexts. Nat. Commun..

[9] Dubuc T.Q., Traylor-Knowles N., Martindale M.Q. (2014). Initiating a regenerative response; cellular and molecular features of wound healing in the cnidarian Nematostella vectensis. BMC Biol..

[10] Birnbaum K.D., Sánchez Alvarado A. (2008). Slicing across kingdoms: Regeneration in plants and animals. Cell.

[11] Pan X., Zhao Y., Li Y., Chen J., Zhang W., Yang L., Xiong Y.Z., Ying Y., Xu H., Zhang Y. (2024). Mitochondrial dynamics govern whole-body regeneration through stem cell pluripotency and mitonuclear balance. Nat. Commun..

[12] Heleven M., Molina M.D., Jaenen V., Bijnens K., Cebrià F., Smeets K. (2026). Redox-modulated ERK dynamics support wound-dependent tissue formation during early planarian regeneration. iScience.

[13] Xu S., Chisholm A.D. (2014). C. elegans Epidermal Wounding Induces a Mitochondrial ROS Burst that Promotes Wound Repair. Dev. Cell.

[14] Love N.R., Chen Y., Ishibashi S., Kritsiligkou P., Lea R., Koh Y., Gallop J.L., Dorey K., Amaya E. (2013). Amputation-induced reactive oxygen species are required for successful Xenopus tadpole tail regeneration. Nat. Cell Biol..

[15] Mandal A., Pinter K., Drerup C.M. (2018). Analyzing Neuronal Mitochondria in vivo Using Fluorescent Reporters in Zebrafish. Front. Cell Dev. Biol..

[16] Greenspan L.J., Cisneros I., Weinstein B.M. (2024). Dermal Dive: An Overview of Cutaneous Wounding Techniques in Zebrafish. J. Invest. Dermatol..

[17] Zhang M., Li R., Fu S., Kumar S., McGinty J., Qin Y., Chen L. (2025). Deep Learning Enhanced Light Sheet Fluorescence Microscopy for in Vivo 4D Imaging of Zebrafish Heart Beating. Light Sci Appl..

[18] Tanaka E.M., Reddien P.W. (2011). The Cellular Basis for Animal Regeneration. Dev. Cell.

[19] Cebrià F., Adell T., Saló E. (2018). Rebuilding a planarian: from early signaling to final shape. Int. J. Dev. Biol..

[20] Gentile L., Cebrià F., Bartscherer K. (2011). The planarian flatworm: an in vivo model for stem cell biology and nervous system regeneration. Dis. Model. Mech..

[21] Sánchez Alvarado A., Newmark P.A. (1999). Double-stranded RNA specifically disrupts gene expression during planarian regeneration. Proc. Natl. Acad. Sci. USA.

[22] Hall R.N., Weill U., Drees L., Leal-Ortiz S., Li H., Khariton M., Chai C., Xue Y., Rosental B., Quake S.R. (2022). Heterologous reporter expression in the planarian Schmidtea mediterranea through somatic mRNA transfection. Cell Rep. Methods.

[23] Hall R.N., Li H., Chai C., Vermeulen S., Bigasin R.R., Song E.S., Sarkar S.R., Gibson J., Prakash M., Fire A.Z., Wang B. (2024). A genetic and microscopy toolkit for manipulating and monitoring regeneration in Macrostomum lignano. Cell Rep..

[24] Emili E., Pérez-Posada A., Vanni V., Salamanca-Díaz D., Ródriguez-Fernández D., Christodoulou M.D., Solana J. (2025). Allometry of cell types in planarians by single-cell transcriptomics. Sci. Adv..

[25] Davidian D., Ziman B., Escobar A.L., Oviedo N.J. (2021). Direct Current Electric Stimulation Alters the Frequency and the Distribution of Mitotic Cells in Planarians. Bioelectricity.

[26] Beane W.S., Adams D.S., Morokuma J., Levin M. (2019). Live imaging of intracellular pH in planarians using the ratiometric fluorescent dye SNARF-5F-AM. Biol. Methods Protoc..

[27] Stevenson C.G., Beane W.S. (2010). A Low Percent Ethanol Method for Immobilizing Planarians. PLoS One.

[28] Bohr T.E., Shiroor D.A., Adler C.E. (2021). Planarian stem cells sense the identity of the missing pharynx to launch its targeted regeneration. eLife.

[29] Talbot J., Schötz E.M. (2011). Quantitative characterization of planarian wild-type behavior as a platform for screening locomotion phenotypes. J. Exp. Biol..

[30] Jaenen V., Bijnens K., Heleven M., Artois T., Smeets K. (2023). Live Imaging in Planarians: Immobilization and Real-Time Visualization of Reactive Oxygen Species. Methods Mol. Biol..

[31] Shen W., Shen Y., Lam Y.W., Chan D. (2018). Live Imaging of Planaria. Methods Mol. Biol..

[32] Dexter J.P., Tamme M.B., Lind C.H., Collins E.M.S. (2014). On-chip immobilization of planarians for in vivo imaging. Sci. Rep..

[33] Goel T., Wang R., Martin S., Lanphear E., Collins E.-M.S. (2019). Linalool acts as a fast and reversible anesthetic in Hydra. PLoS One.

[34] Lee J.-R., Boothe T., Mauksch C., Thommen A., Rink J.C. (2024). Epidermal turnover in the planarian Schmidtea mediterranea involves basal cell extrusion and intestinal digestion. Cell Rep..

[35] Vila-Farré M., Rozanski A., Ivanković M., Cleland J., Brand J.N., Sandberg F., Grohme M.A., von Kannen S., Grosbusch A.L., Vu H.T.-K. (2023). Evolutionary dynamics of whole-body regeneration across planarian flatworms. Nat. Ecol. Evol..

[36] Marchant J.S. (2019). Ca2+ Signaling and Regeneration. Cold Spring Harb. Perspect. Biol..

[37] Tu M.K., Borodinsky L.N. (2014). Spontaneous calcium transients manifest in the regenerating muscle and are necessary for skeletal muscle replenishment. Cell Calcium.

[38] Qiao H.-H., Wang F., Xu R.-G., Sun J., Zhu R., Mao D., Ren X., Wang X., Jia Y., Peng P. (2018). An efficient and multiple target transgenic RNAi technique with low toxicity in Drosophila. Nat. Commun..

[39] Persico A., Molteni L., Mantecca P., Kravicz M., Bragato C. (2025). Transgenic zebrafish embryos to evaluate the in vivo effects of different liposome-paclitaxel nanocarrier system. Sci. Rep..

[40] Bouffard J., Asthagiri A.R., Cram E.J. (2014). In vivo monitoring of calcium signaling at the single-cell and tissue levels in the spermatheca of C. elegans. Mol. Biol. Cell.

[41] Nasufovic V., Kompa J., Lindamood H.L., Blümke M., Dibsy R., Koch B., Levario-Diaz V., Weber K., Maager M., Nomerotskaia E. (2025). SiR-XActin: A Fluorescent Probe for Imaging Actin Dynamics in Live Cells. Angew. Chem. Int. Ed. Engl..

[42] Xu K., Qiang M., Gao W., Su R., Li N., Gao Y., Xie Y., Kong F., Tang B. (2013). A near-infrared reversible fluorescent probe for real-time imaging of redox status changes in vivo. Chem. Sci..

[43] Ortega-Villasante C., Burén S., Blázquez-Castro A., Barón-Sola Á., Hernández L.E. (2018). Fluorescent in vivo imaging of reactive oxygen species and redox potential in plants. Free Radic. Biol. Med..

[44] Jaenen V., Fraguas S., Bijnens K., Heleven M., Artois T., Romero R., Smeets K., Cebrià F. (2021). Reactive oxygen species rescue regeneration after silencing the MAPK-ERK signaling pathway in Schmidtea mediterranea. Sci. Rep..

[45] Zhong R., Wang X., Li H., Zhang Q. (2026). Regeneration-on-a-chip: a planarian microfluidic device enabling automated cultivation, individual tracking and *in vivo* imaging for regeneration study. Analyst.

[46] Taheri Mirghaed A., Ghelichpour M., Hoseini S.M. (2016). Myrcene and linalool as new anesthetic and sedative agents in common carp, Cyprinus carpio - Comparison with eugenol. Aquaculture.

[47] Szaszkiewicz J., Leigh S., Hamilton T.J. (2021). Robust behavioural effects in response to acute, but not repeated, terpene administration. Sci. Rep..

[48] Bijnens K., Jaenen V., Wouters A., Leynen N., Pirotte N., Artois T., Smeets K. (2021). A Spatiotemporal Characterisation of Redox Molecules in Planarians, with a Focus on the Role of Glutathione during Regeneration. Biomolecules.

[49] Leal-Cardoso J.H., da Silva-Alves K.S., Ferreira-da-Silva F.W., dos Santos-Nascimento T., Joca H.C., de Macedo F.H.P., de Albuquerque-Neto P.M., Magalhães P.J.C., Lahlou S., Cruz J.S. (2010). Linalool blocks excitability in peripheral nerves and voltage-dependent Na+ current in dissociated dorsal root ganglia neurons. Eur. J. Pharmacol..

[50] Hashimoto M., Takahashi K., Ohta T. (2023). Inhibitory effects of linalool, an essential oil component of lavender, on nociceptive TRPA1 and voltage-gated Ca2+ channels in mouse sensory neurons. Biochem. Biophys. Rep..

[51] Phuong T.N.T., Jang S.H., Rijal S., Jung W.K., Kim J., Park S.J., Han S.K. (2021). GABA- and Glycine-Mimetic Responses of Linalool on the Substantia Gelatinosa of the Trigeminal Subnucleus Caudalis in Juvenile Mice: Pain Management through Linalool-Mediated Inhibitory Neurotransmission. Am. J. Chin. Med..

[52] Poyton C., Manchadi M.-L., Cheesman M., Lavidis N. (2015). Effects of Lavender and Linalool on Neurotransmission and Contraction of Smooth Muscle. Phcog. Commn..

[53] Milanos S., Elsharif S.A., Janzen D., Buettner A., Villmann C. (2017). Metabolic Products of Linalool and Modulation of GABAA Receptors. Front. Chem..

[54] Bäcktorp C., Wass J.R.T.J., Panas I., Sköld M., Börje A., Nyman G. (2006). Theoretical Investigation of Linalool Oxidation. J. Phys. Chem. A.

[55] Lowe J.R., Mahool T.D., Staehle M.M. (2015). Ethanol exposure induces a delay in the reacquisition of function during head regeneration in Schmidtea mediterranea. Neurotoxicol. Teratol..

[56] Inoue T., Kumamoto H., Okamoto K., Umesono Y., Sakai M., Sánchez Alvarado A., Agata K. (2004). Morphological and Functional Recovery of the Planarian Photosensing System during Head Regeneration. Zoolog. Sci..

[57] Rink J.C., Vu H.T.K., Sánchez Alvarado A. (2011). The maintenance and regeneration of the planarian excretory system are regulated by EGFR signaling. Development.

