# Supplementary material for: Linalool enables reversible immobilization for high-resolution in vivo imaging during regeneration in Schmidtea mediterranea
Source: iScience. 2026 Sep 4;29(9):117282. doi: 10.1016/j.isci.2026.117282 (PMC13571967; doi:10.1016/j.isci.2026.117282)
Supplement: Supplementary file 1 [file mmc1.pdf]

iScience, Volume *29*

Supplemental information

Linalool enables reversible immobilization for high-resolution *in vivo* imaging during regeneration in *Schmidtea mediterranea*

Martijn Heleven, Charlotte Segnana, Bob Kauffmann, Reja Trippaers, Karolien Bijnens, and Karen Smeets

Supplemental figures

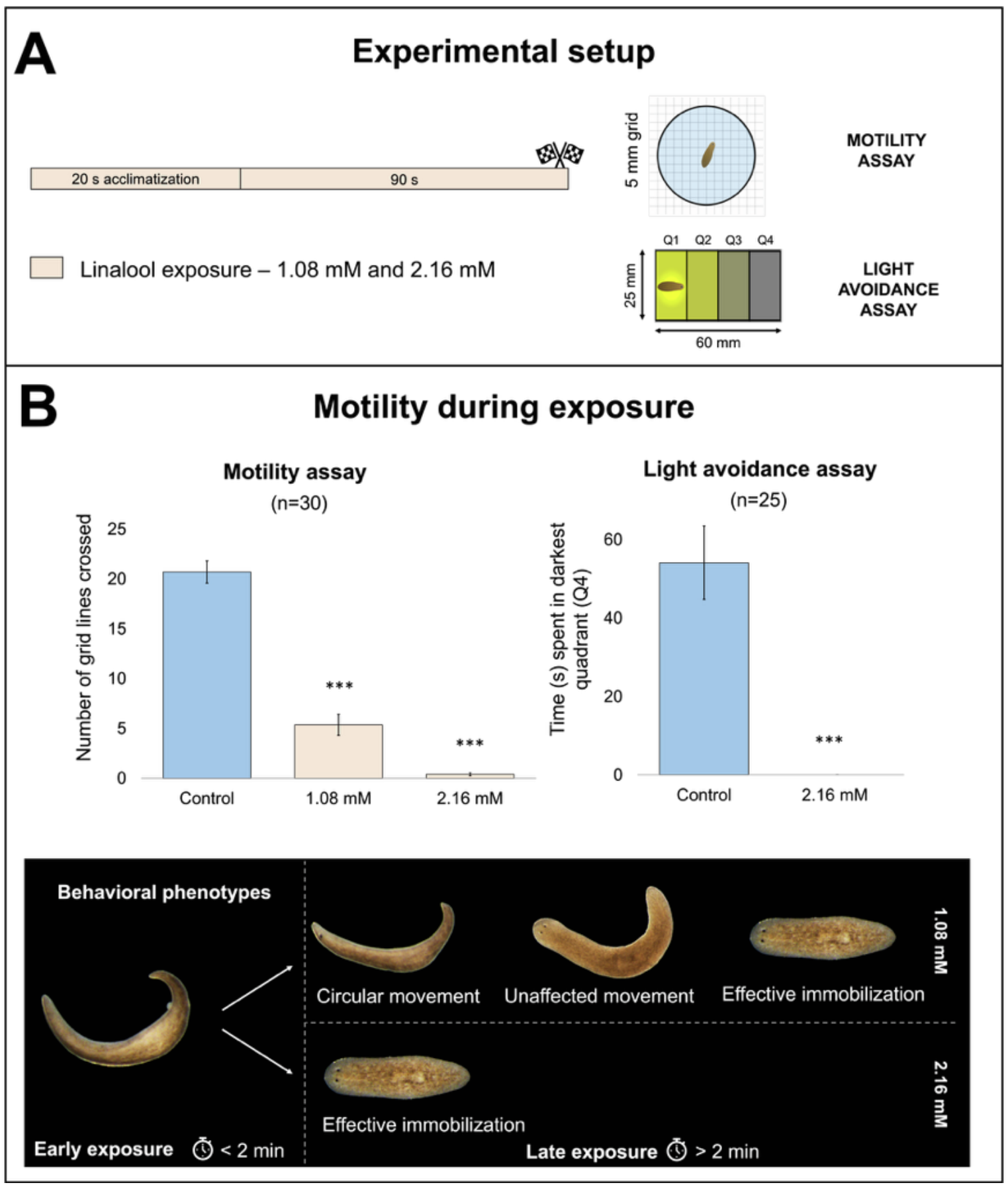

Supplementary figure 1: 2.16 mM linalool effectively impairs motility A) Graphical representation of the experimental setup. Animals were exposed to either medium, 1.08 mM or 2.16 mM linalool. Following 2 min of linalool exposure, animals were transferred to the assay setup and allowed to acclimatize for 20 s under continued exposure. Motility was then assessed for 90 s using either the motility or light avoidance test. In the light avoidance test the time spent in the darkest quadrant was quantified, while the number of grid lines crossed was quantified in the motility assay. **B)** Motility quantification during linalool exposure. Performance during the motility assay was quantified for both concentrations (n = 30 per condition). Subsequently, the effect of 2.16 mM linalool on the capacity to move in a light-induced gradient was studied (n = 25 per condition). Behavioral phenotypes that could be observed include curling during early exposure (<2 min). After 2 min, animals exposed to 1.08 mM linalool displayed circular movement, unaffected movement, or effective immobilization. Exposure to 2.16 mM linalool effectively immobilized 100% of the animals. Data represent mean ± SEM from three independent experiments. Statistical significance was assessed using Wilcoxon rank sum test with Holm correction for multiple comparisons, and indicated by *: $p < 0.05$, **: $p < 0.01$, ***: $p < 0.001$.

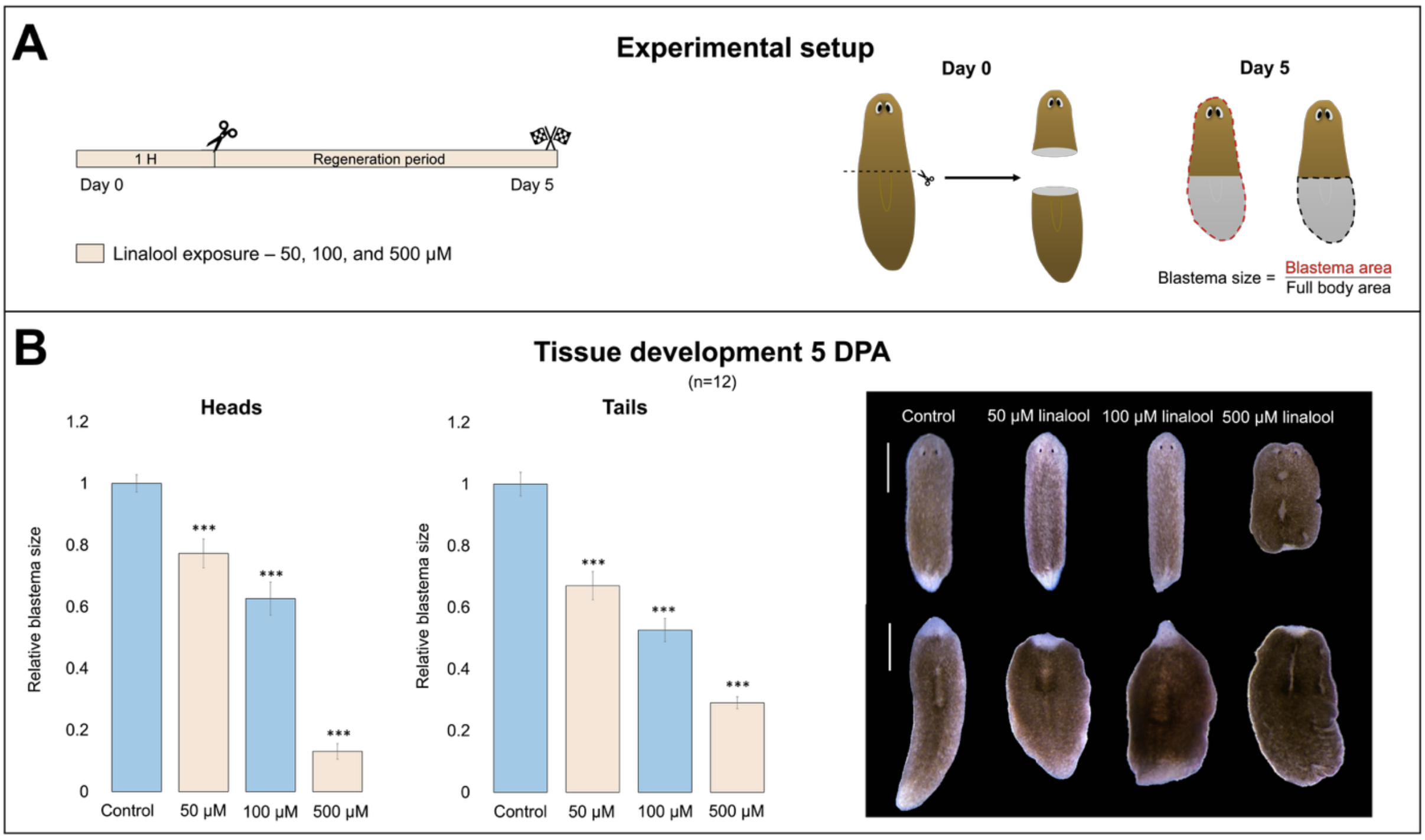

Supplementary figure 2: Continuous linalool exposure impairs regeneration. **A)** Graphical representation of the experimental setup. Animals were pre-exposed for 1 h to either medium, 50, 100, or 500 µM linalool, after which they were transversely amputated anterior to the pharynx and allowed to regenerate during continuous exposure. At 5 DPA, tissue development was scored by measuring the relative blastema size, which corresponds to the ratio of the total blastema area to the full body area. **B)** Tissue development at 5 DPA (n = 12 per condition). Graphs represent the relative blastema size of regenerating heads and tails compared to the control. Representative images of regenerating heads and tails are shown for each exposure concentration. Scale bars, 1000 µm. Data represent mean ± SEM from two independent experiments. Statistical significance compared to the control was assessed using unpaired Student's *t* tests with Holm correction for multiple comparisons, indicated by *: $p < 0.05$, **: $p < 0.01$, ***: $p < 0.001$.

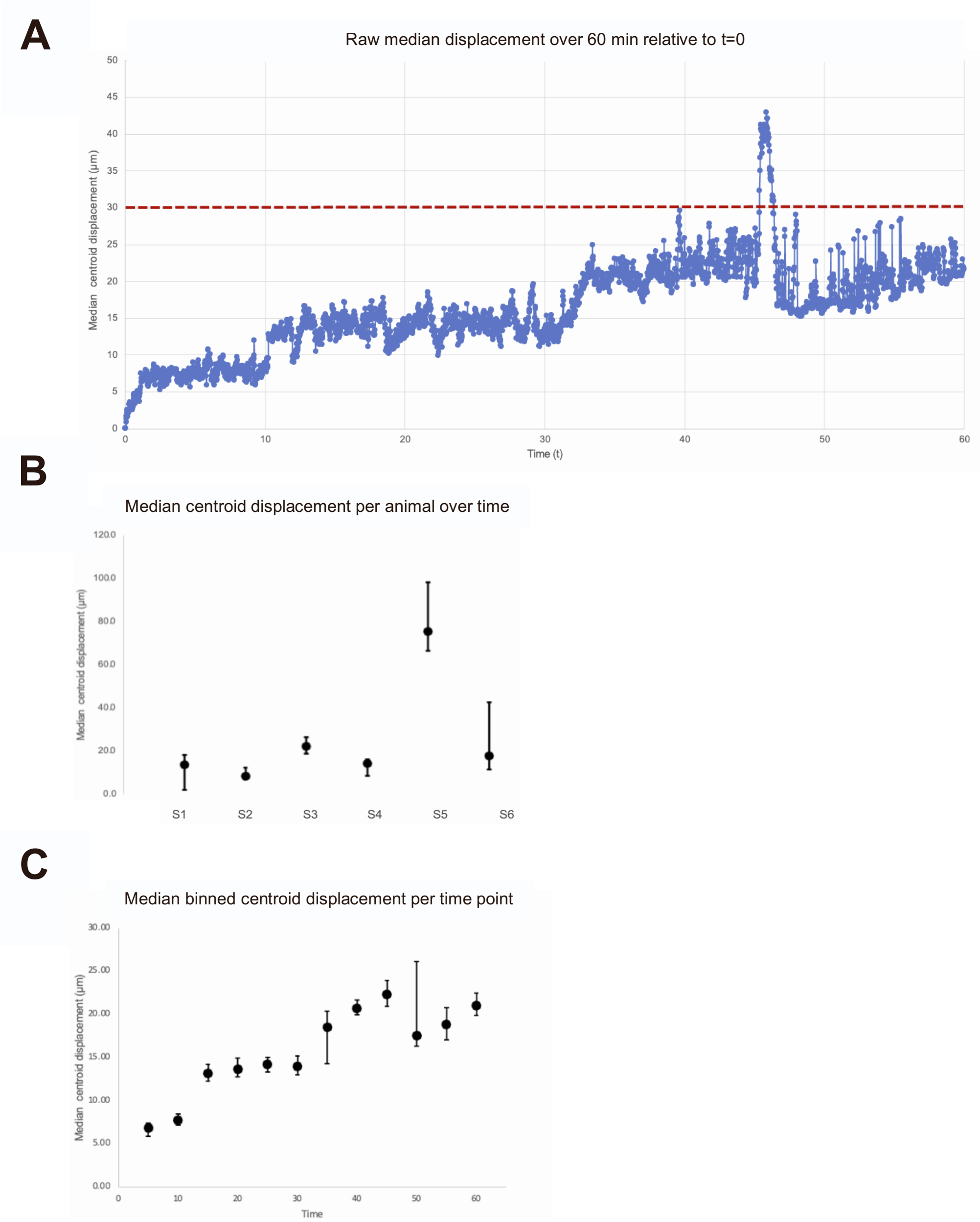

Supplementary figure 3: Linalool allows stable immobilization for real-time *in vivo* imaging. A) Raw median displacement (µm) over 60 min relative to the position at t = 0 min. **B)** Median centroid displacement per animal over time (µm). **C)** Median binned displacement per time point (µm), data were binned into 10 min intervals. For B) and C), points and error bars indicate the median and interquartile range (IQR), respectively. Data were obtained from six independent animals.

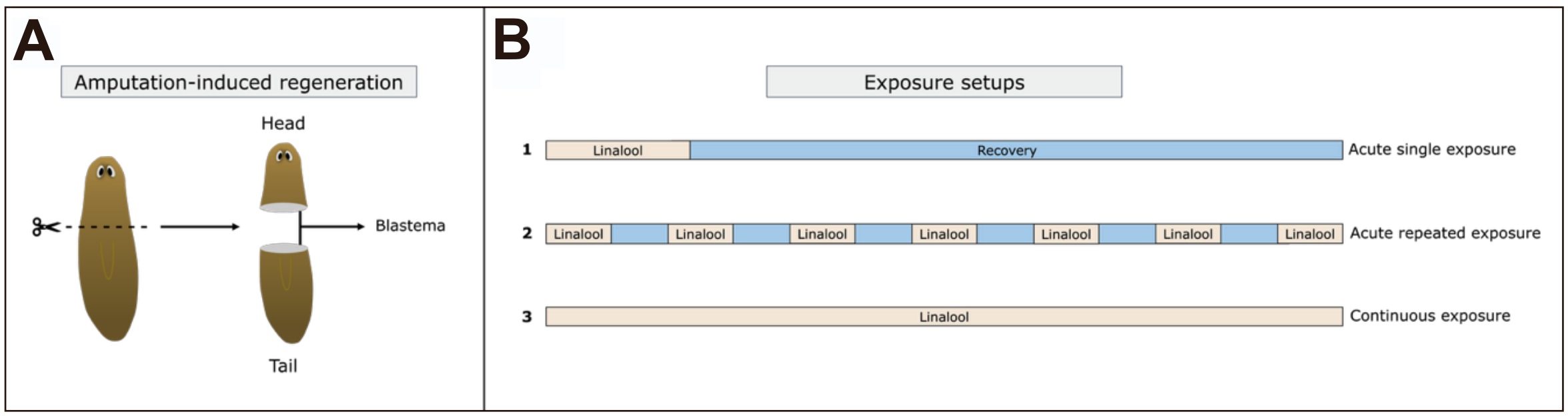

Supplementary figure 4: General experimental setup. A) Amputation-induced regeneration setup. Intact planarians were amputated anterior to the pharynx to generate a regenerating head and tail. **B)** Exposure setups used throughout the study: (1) acute single exposure, (2) acute repeated exposure, and (3) continuous exposure. Color code: pink= linalool exposure; blue= recovery in medium.

Supplemental tables

Table S1: Linalool-compatible dyes and probes for *in vivo* imaging

Dye	Ex/Em	Target	Manufacturer	Working concentration
ROS-ID® Superoxide detection kit	550/620 nm	Superoxide	Enzo (51012)	25 µM
Peroxy Orange 1 (PO1)	543/565 nm	Hydrogen peroxide	Sigma-Aldrich (SML0688)	20 µM
CellROX Orange	545/565 nm	Reactive oxygen species	Fisher Scientific (12807322)	10 µM
CellROX Deep Red	652/669 nm	Reactive oxygen species	Invitrogen (12013765)	30 µM
CellMask Green Plasma Membrane Stain	522/535 nm	Plasma membrane	Invitrogen (C37608)	1:1000 dilution of 1000X stock
MitoTracker Red CMXRos	579/599 nm	Active mitochondria	Invitrogen (11569106)	5 µM
SYTO 61	628/645 nm	DNA	Thermo Fisher Scientific (S11343)	2.5 µM
DAPI	341/452 nm	DNA	Invitrogen (11580246)	16 µM
Hoechst 33342	405/440 nm	DNA	Thermo Fisher Scientific (H3570)	8 µM
